# Identification of differential PI3K pathway target dependencies in T-cell acute lymphoblastic leukemia through a large cancer cell panel screen

**DOI:** 10.18632/oncotarget.8031

**Published:** 2016-03-10

**Authors:** James T. Lynch, Robert McEwen, Claire Crafter, Ultan McDermott, Mathew J. Garnett, Simon T. Barry, Barry R. Davies

**Affiliations:** ^1^ Oncology iMED, AstraZeneca, Alderley Park, Macclesfield, SK10 4TG, United Kingdom; ^2^ Wellcome Trust Sanger Institute, Wellcome Trust Genome Campus, Hinxton, Cambridgeshire, CB10 1SA, United Kingdom

**Keywords:** PI3K, AKT, mTOR, Notch, leukemia

## Abstract

Selective phosphoinositide 3-kinase (PI3K)/AKT/mTOR inhibitors are currently under evaluation in clinical studies. To identify tumor types that are sensitive to PI3K pathway inhibitors we screened compounds targeting PI3Kα/δ (AZD8835), PI3Kβ/δ (AZD8186), AKT (AZD5363) and mTORC1/2 (AZD2014) against a cancer cell line panel (971 cell lines). There was an enrichment of hematological malignancies that were sensitive to AKT and mTOR inhibition, with the greatest degree of sensitivity observed in T-cell acute lymphoblastic leukemia (T-ALL). We found that all NOTCH mutant T-ALL cell lines were sensitive to AKT and mTORC1/2 inhibitors, with only partial sensitivity to agents that target the PI3K α, β or δ isoforms. Induction of apoptosis only occurred following AKTi treatment in cell lines with PTEN protein loss and high levels of active AKT. In summary, we have demonstrated that T-ALL cell lines show differential sensitivity to inhibition at different nodes in the PI3K/AKT/mTOR pathway and inhibiting AKT or mTOR may have a therapeutic benefit in this disease setting.

## INTRODUCTION

T-cell acute lymphoblastic leukemia (T-ALL) is the most common form of malignancy in children and is classified by the abnormal accumulation of immature lymphoblasts of the T-cell lineage [1]. T-ALL accounts for 10–15% of pediatric and 25% of adult ALL. Activating mutations in *NOTCH1* are present in 55–60% of T-ALL and mutations in *PTEN* and the phosphoinositide 3-kinase (PI3K) pathway are present in 47% of pediatric cases [2–5]. The discovery of these recurring mutations has led to pre-clinical studies investigating the potential for using NOTCH and PTEN pathway agents in T-ALL. With respect to PI3K pathway inhibitors, it is not clear what the optimal strategy is to effectively target T-ALL survival [4, 6].

The PI3K/AKT/mTOR signaling pathway is the most frequently activated signaling network in cancer and recurring mutations in this network have been identified, including mutations (*PIK3CA*, *AKT*, and *PTEN*), amplifications (*PIK3CA*, *PIK3CB* and *AKT*) and deletions (*PTEN*, *INPP4B*) [7–12]. The high frequency of pathway activation and the identification of recurring mutations in cancer has led to the intensive development of PI3K pathway inhibitors [10]. Studies have demonstrated that tumors with loss of the tumor suppressor *PTEN*, a PIP_3_ phosphatase, display increased sensitivity to PI3Kβ and AKT inhibitors [13–15]. In contrast, cell lines with *PIK3CA* mutations generally do not respond to PI3Kβ inhibitors but encouraging results have been observed with PI3Kα and AKT inhibitors [16, 17].

The development of selective PI3K pathway inhibitors allows us to better understand intrinsic target dependencies in the PI3K pathway to determine the optimal strategy for pathway inhibition. In addition, advances in screening technologies have enabled investigation of inhibitor sensitivity across large panels of cell lines spanning multiple cancer types [18]. To this end, we sought to identify novel disease settings that display differential sensitivity to PI3K pathway inhibitors across a large cancer cell line panel.

## RESULTS

### Sensitivity to PI3K/AKT/mTOR pathway inhibitors in a large cancer cell line panel

**Figure 1:**
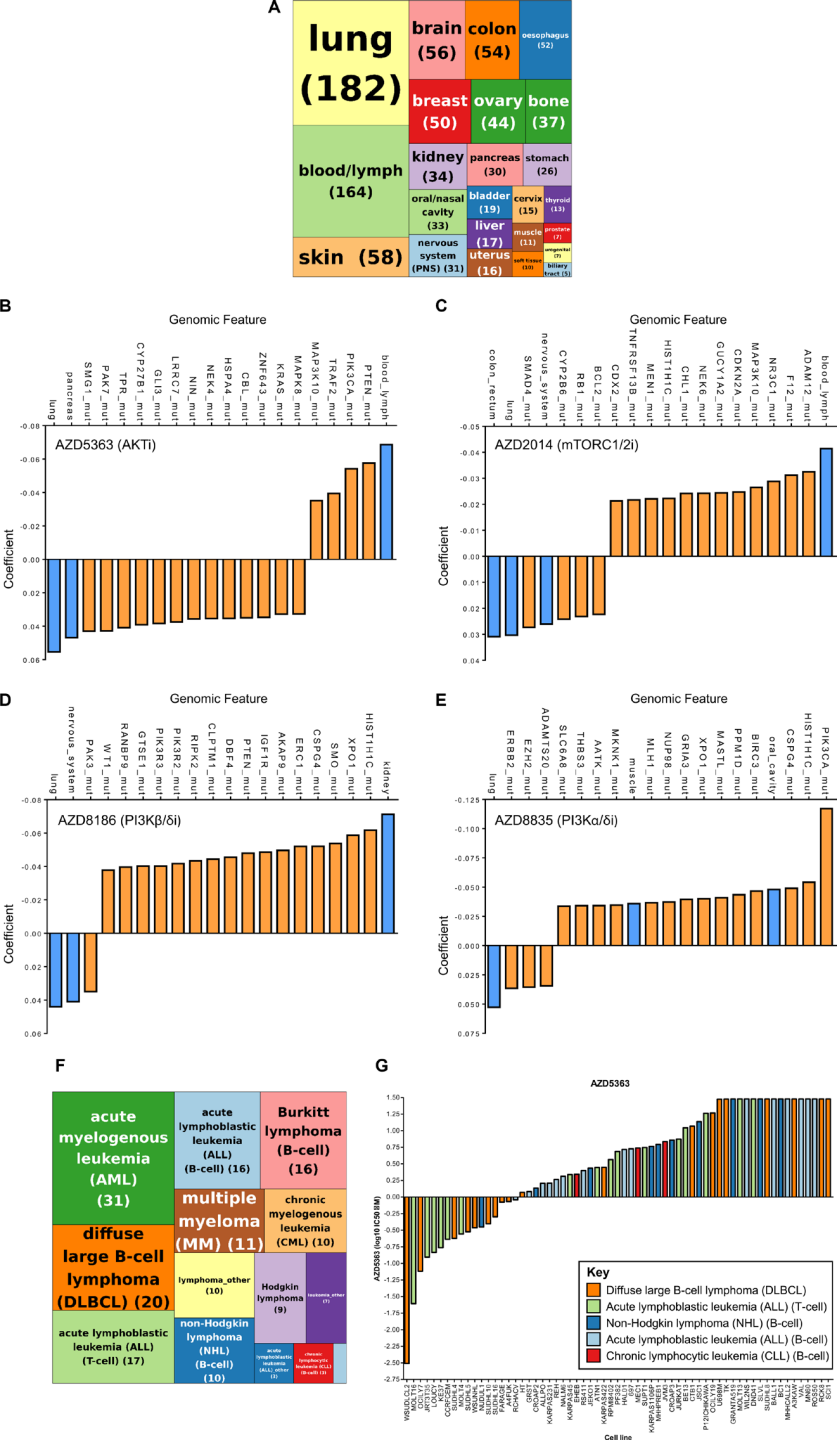
A cancer cell line pharmacology screen of PI3K pathway inhibitors identified strong activity of AKT and mTORC1/2 inhibitors in hematological malignancies 971 cancer cell lines were screened against four PI3K pathway inhibitors and IC_50_ values determined. (A) Tree map depicting the distribution of 971 cancer cell lines across 24 lineages. Figures in parentheses represent the numbers of cell lines. (B–E) Elastic net regularization analysis for AZD5363 (B), AZD2014 (C), AZD8186 (D) and AZD8835 (E). The coefficient value signifies the correlation between sensitivity (IC_50_ value) and the input variable (e.g. genetic aberration/lineage) based on elastic net regression analysis, with a larger positive or negative coefficient representing a more significant correlation. Blue bars represent lineage input variables and orange bars represent genetic aberration input variables. (F) Tree map depicting the distribution of 164 hematological cancer cell lines across different hematological subtypes. Pale blue box represents “Myeloma_other” (1). Figures in parentheses represent the numbers of cell lines. (G) Waterfall plot of T-cell acute lymphoblastic leukemia (T-ALL), B-cell acute lymphoblastic leukemia (B-ALL), chronic lymphocytic leukemia (CLL), diffuse large B-cell lymphoma (DLBCL) and non-Hodgkin lymphoma (NHL) cancer cell lines ordered according to sensitivity to AZD5363.

To further understand the contribution of the different nodes in the PI3K pathway, we used four kinase inhibitors that are currently in clinical development, AZD8835 a PI3Kα and PI3Kδ inhibitor (PI3Kα/δi); AZD8186 a PI3Kβ and PI3Kδ inhibitor (PI3Kβ/δi); AZD5363 an pan-AKT inhibitor (AKTi) and AZD2014 an mTORC1/2 inhibitor (mTORC1/2i) [13, 16, 19, 20]. To identify novel disease settings that display sensitivity to the PI3K pathway inhibitors, the compounds were profiled across a large cancer cell line panel [18]. The cell line panel consisted of 971 cell lines spanning 24 tissue types (Figure 1A). IC_50_ values were generated from 72 hour proliferation assays using a nine-point concentration range for each compound. The complete screening results can be found in Supplementary Table 1.

In order to interrogate the large datasets we performed elastic net regularization [21]. There is a strong correlation between AKTi sensitive cell lines and cell lines that have mutations in either the *PTEN* gene (loss of function/deletion mutations) or *PIK3CA* mutations, which is in keeping with previously published data (Figure 1B) [16]. Interestingly, cell lines that belong to the blood/lymph lineage also display increased sensitivity to AKTi. Cell lines with mutations in the *KRAS* oncogene and lung/pancreatic lineages were associated with resistance to AKTi, a feature which has been previously described [16].

For mTORC1/2i, cell lines that displayed sensitivity to this compound and the strongest association with a predictor variable was correlation with sensitivity in the blood/lymph lineage (Figure 1C). Similar observations have been previously described and the effectiveness of mTOR inhibitors are currently being investigated in a number of hematological malignancies [22].

Studies have demonstrated that cells that are deficient for *PTEN* function are sensitive to PI3Kβ inhibitors, although the mechanism by which *PTEN* loss confers PI3Kβ dependency is still unknown [13–15]. Our data supports this correlation and cell lines with *PTEN* mutations were associated with sensitivity to PI3Kβ/δi (Figure 1D). Similar to AKTi, *NRAS* mutant cell lines negatively associate with the PI3Kβ/δi response.

For PI3Kα/δi, the elastic net analysis demonstrated a strong association between sensitivity and *PIK3CA* mutations, suggesting that the compound is selectively inhibiting *PIK3CA* mutant cell lines (Figure 1E).

In summary, a PI3K pathway inhibitor cancer cell line screen identified the hematological malignancy subtype as being the most sensitive to AKT and mTOR inhibition. In addition, there are a number of other predictor variables, both genomic features and lineage that associate with the sensitivity of these compounds.

### Activity of PI3K pathway inhibitors in hematological malignancies

AKTi and mTORC1/2i were more active in the blood/lymph lineage. To investigate this observation in greater detail we classified the hematological malignancies into their lymphoma/leukemia subtypes (Figure 1F and Supplementary Table 2). We focused on the AKTi and used a sensitivity cut-off of 9 μM, which approximately classified the same cell lines as being sensitive to AKTi as previously described [16]. We found a number of hematological subtypes with IC_50_ values < 9 μM in greater than 50% of the cell lines profiled, including T-ALL, B-cell acute lymphoblastic leukemia (B-ALL), chronic lymphocytic leukemia (CLL), diffuse large B-cell lymphoma (DLBCL) and non-Hodgkin lymphoma (NHL) (Figure 1G and Table 1). T-ALL was selected for further investigation as it demonstrated the highest degree of sensitivity (71%) in subtypes where there were > 10 cell lines that were sensitive to AKTi.

**Table 1:** Sensitivity of the hematological malignancy subtypes to AZD5363

Subtype	Total number of cell lines	AZD5363 sensitive (< 9 μmol/L)	% AZD5363 sensitive (< 9 μmol/L)
Acute myelogenous leukemia (AML)	31	15	48
Acute lymphoblastic leukemia (ALL) (T-cell)	17	12	71
Diffuse large B-cell lymphoma (DLBCL)	20	11	55
Acute lymphoblastic leukemia (ALL) (B-cell)	16	9	56
Non-Hodgkin lymphoma (NHL) (B-cell)	10	6	60
Burkitt lymphoma (B-cell)	16	5	31
Chronic myelogenous leukemia (CML)	10	5	50
Multiple myeloma (MM)	11	4	36
Chronic lymphocytic leukemia (CLL) (B-cell)	3	3	100
Hodgkin lymphoma	9	3	33
Leukemia_other	7	3	43
Lymphoma_other	10	3	30
Acute lymphoblastic leukemia (ALL)_other	3	1	33
Myeloma_other	1	0	0
**Total**	**164**	**81**	**49**

Hematological cancer cell lines were categorised by subtype and the values represent the total number of cell lines in each subtype (left column), the number of cell lines in that subtype that are sensitive to AZD5363 (< 9 μM) (middle panel) and the percentage of cell lines in that subtype that are sensitive to AZD5363 (< 9 μM) (right column).

**Figure 2:**
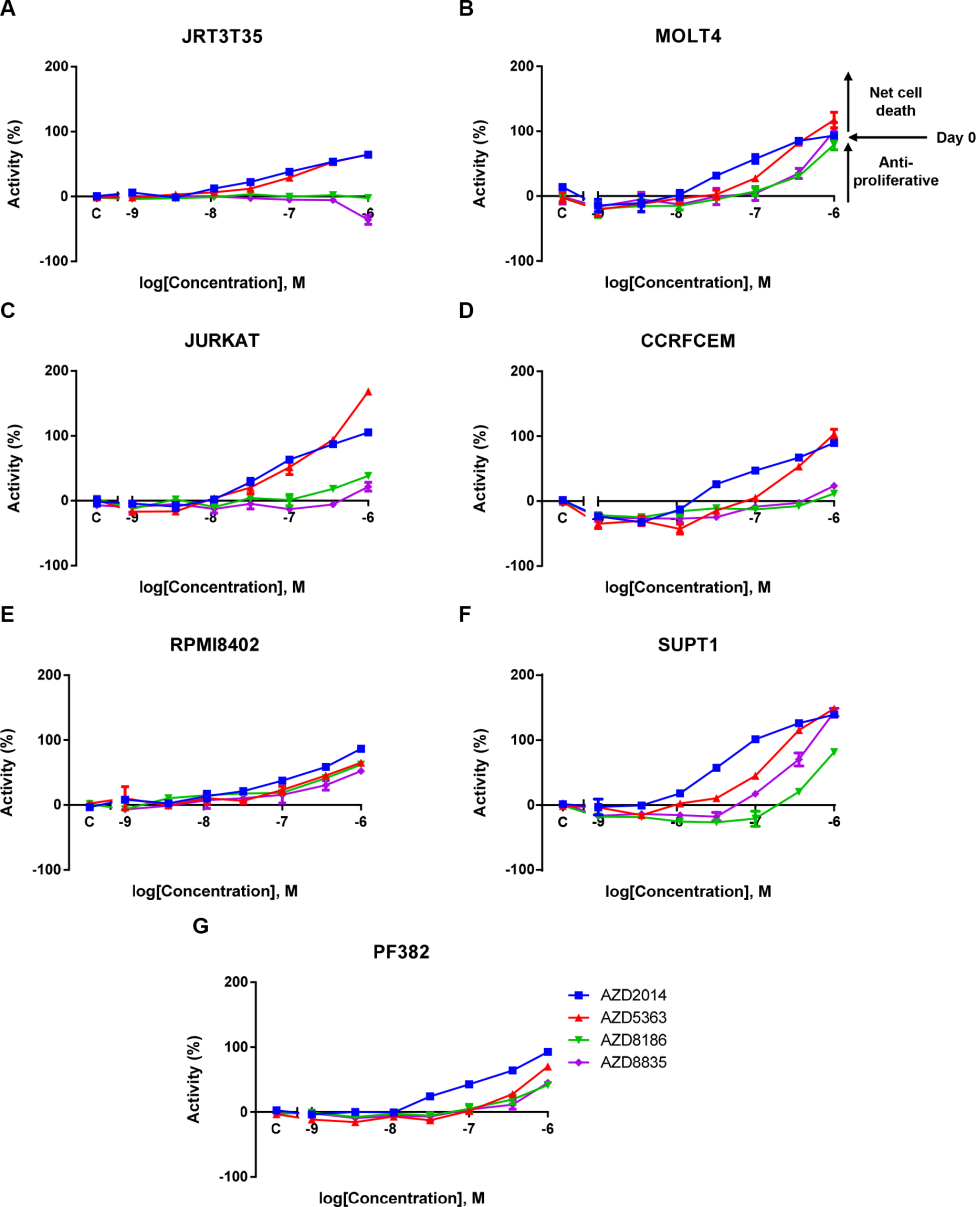
A validation T-ALL screen confirms AKT and mTORC1/2 as key mediators of T-ALL cell proliferation and survival A panel of T-ALL cell lines were treated with a dose response of four PI3K pathway inhibitors. Live cell number was assessed after five days using a sytox green endpoint. (**A–G**) Exemplar mean ± S.E.M. dose response curves for the four inhibitors across the T-ALL cell line panel. 100% activity represents the initial seeding density on the day of dosing (Day 0) (*n* = 2). S.E.M = standard error of the mean.

Given that adherent and suspension cell lines were screened using different assay formats in the cancer cell line panel, we also wanted to determine whether T-ALL cell lines display enhanced sensitivity or whether this was an artefact of the different screening methodologies. To do this we performed a validation screen using an Acumen Explorer based sytox green assay, optimised for suspension cells, which is a method similar to the adherent cell line screening. We selected seven T-ALL cell lines from the original screen that had a AKTi IC_50_ value < 9 μM, ranging from 0.12 μM to 7.5 μM (Table 2). We generated dose response curves and IC_50_ values (Figure 2 and Table 2). All T-ALL cell lines have an IC_50_ value < 1 μM following treatment with either AKTi or mTORC1/2i, whereas there was a varying degree of sensitivity to the PI3K inhibitors. In keeping with the role of PI3Kβ in *PTEN* deficient tumors, of the three cell lines that were sensitive to PI3Kβ/δi, two have *PTEN* deletions [14, 15, 23]. There were two other *PTEN* null cell lines that were not sensitive to PI3Kβ/δi, although they were sensitive to AKTi. The SUPT1 cell line displayed the strongest sensitivity to PI3Kα/δi, with this cell line harbouring an activating mutation in PIK3CA [23].

**Table 2:** Sensitivity of PI3K pathway inhibitors in T-ALL

		Cell Line						
		JRT3T35	CCRFCEM	MOLT4	RPMI8402	PF382	SUPT1	JURKAT
Mutation status	PI3KCA	WT	WT	WT	WT	WT	MUT	WT
	PTEN	LOF.DEL	LOF.DEL	LOF.DEL	LOF.DEL	WT	WT	LOF.DEL
Sanger screen (IC_50_ μM)	AZD2014 (mTORC1/2i)	0.08	0.04	0.05	0.08	4.3	8.98	0.27
	AZD5363 (AKTi)	0.12	0.23	0.28	3.66	4.86	5.56	7.45
	AZD8186 (PI3Kβ/δi)	7.61	> 10	0.53	0.22	> 10	> 10	> 10
	AZD8835 (PI3Kα/δi)	> 10	6.94	0.81	1.1	> 10	> 10	> 10
Validation screen (IC_50_ μM)	AZD2014 (mTORC1/2i)	0.19	0.08	0.39	0.19	0.11	0.03	0.2
	AZD5363 (AKTi)	0.27	0.3	0.28	0.56	0.56	0.1	0.22
	AZD8186 (PI3Kβ/δi)	> 1	> 1	0.89	0.8	> 1	0.64	> 1
	AZD8835 (PI3Kα/δi)	> 1	> 1	0.91	> 1	> 1	0.29	> 1
PI3K pathway inhibitor screen (IC_50_ μM)	GSK690693 (AKTi)	0.11	0.3	0.18	0.16	0.21	0.12	0.17
	GSK2110183 (AKTi)	0.13	0.3	0.36	0.43	0.89	0.4	0.17
	MK2206 (AKTi)	0.23	0.12	0.21	0.8	0.25	0.23	0.24
	CC223 (mTORC1/2i)	0.71	0.68	1.6	0.59	0.7	0.16	0.41
	INK128 (mTORC1/2i)	0.03	0.03	0.15	0.1	0.05	0.01	0.08
	GDC0941 (PAN PI3Ki)	3.3	2.95	0.64	0.38	1.8	0.05	1.6
	SAR260301 (PI3Kβi)	> 10	> 10	> 10	8.5	> 10	> 10	> 10
	BYL719 (PI3Kαi)	7.1	3	2.8	2.3	4	0.46	7.2
	CAL101 (PI3Kδi)	> 10	5.9	5	5.3	> 10	> 10	> 10

Values represent the IC_50_ values generated from the cancer cell line panel screen (upper panel), validation screen (middle panel) and PI3K pathway inhibitor screen (lower panel). The PTEN and PIK3CA mutation status of these cell lines are indicated. WT = Wild Type. MUT = Mutated. LOF.DEL = Loss of Function/Deletion.

To build on these observations we screened a number of other PI3K/AKT/mTOR inhibitors (Table 2). In keeping with the previous screening results a comparable degree of sensitivity was observed for all AKT and mTOR inhibitors (Supplementary Figure 1 and Table 2). Cell lines that were sensitive to the isoform selective PI3K inhibitors were also sensitive to the pan-PI3K inhibitor (GDC0941), supporting the conclusion that PI3K activity is not functionally relevant in all of the T-ALL cell lines screened [24]. Activity of a PI3Kα inhibitor (BYL719) was observed in the PIK3CA mutant cell line, whereas, CAL101, a PI3Kδ inhibitor, did not appear to demonstrate strong sensitivity to any of the T-ALL cell lines [25, 26]. These results reinforce the findings of the large cell line panel and bespoke screens and further dissect the function of the PI3K isoforms in maintaining T-ALL cell survival.

Given that inhibition of PI3K signaling relieves feedback inhibition of the pathway resulting in the reactivation of signaling, we asked whether combining the PI3K pathway inhibitors would be synergistic [27]. To investigate this, we performed an intra-pathway combination screen where each of the four PI3K pathway inhibitors were combined with each other to determine synergistic effects. The combination of any of the pathway agents did not cause strong synergism, as determined by the Loewe model of additivity (Supplementary Figures 2, 3 and Supplementary Table 3). The majority of the synergy scores were less than one and there were no synergy scores greater than five, which is a cut-off used previously to identify combinations of interest in a high throughout screen [28]. Therefore, at concentrations where there is monotherapy activity in a cell line, additive but not synergistic effects were observed.

### Regulation of PI3K pathway signaling in T-ALL

**Figure 3:**
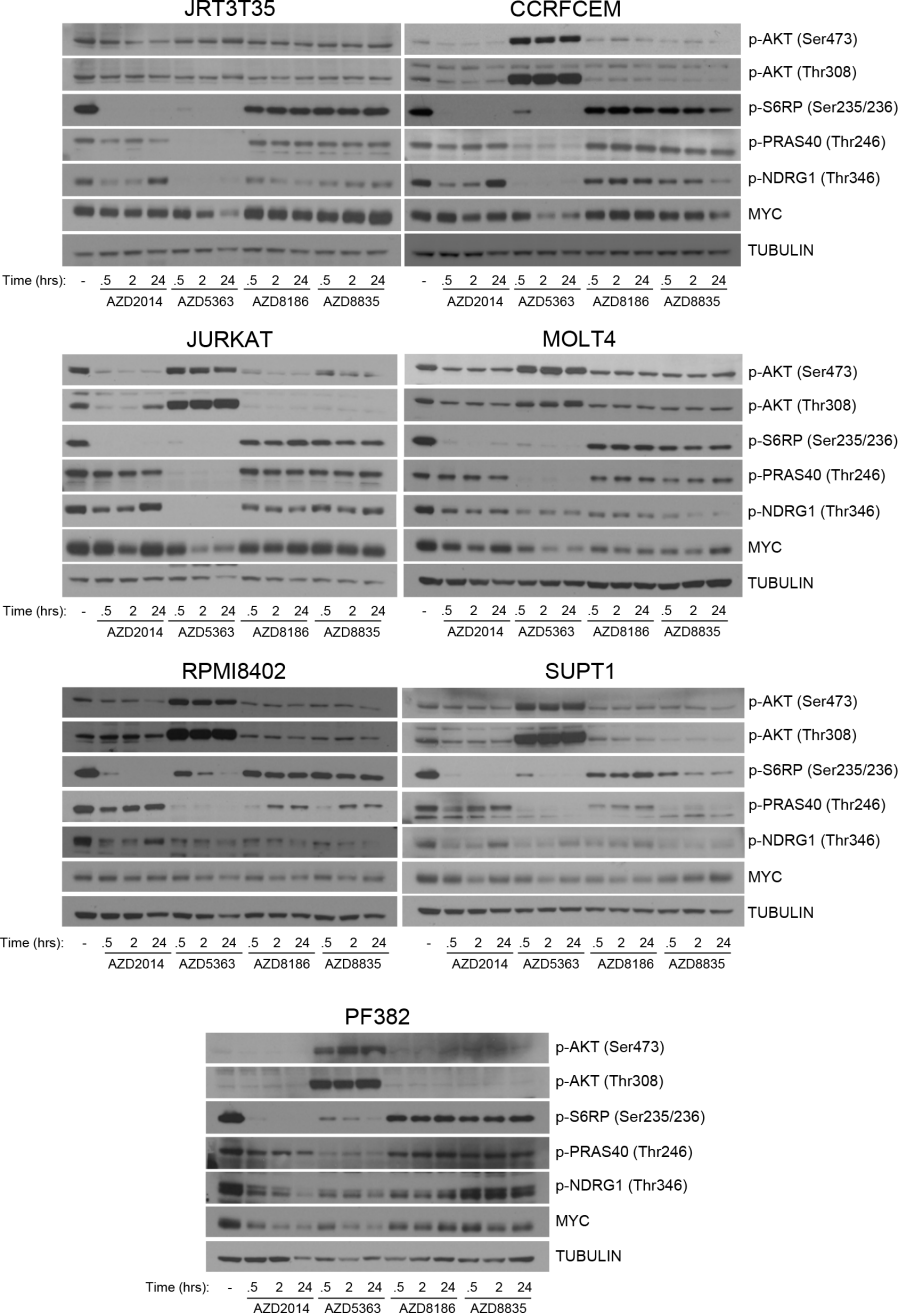
Regulation of downstream signaling following PI3K pathway inhibition in T-ALL cell lines Seven T-ALL cell lines were treated with AZD2014 (0.25 μM), AZD5363 (1 μM), AZD8186 (0.5 μM) and AZD8835 (0.5 μM) for the times indicated. Western blot analysis was performed to determine modulation of the indicated proteins and phosphorylated proteins following pathway inhibition (*n* = 2).

To understand the drivers of this difference between the PI3K pathway inhibitors, downstream signaling in the T-ALL cell lines was determined. All cell lines were treated with an inhibitor time course at concentrations that are either achievable in the clinic (AKTi/mTORC1/2i) or similar to the IC_50_ values of the sensitive cell lines from the screen, whilst maintaining PI3K isoform selectivity (PI3Kβ/δi/PI3Kα/δi) (Supplementary Table 4). Following treatment with AKTi and mTORC1/2i, we observed strong suppression of p-S6RP across all cell lines (Figure 3). Loss of p-NDRG1 was also observed, with varying degrees of reactivation at 24 hours post treatment. AKTi strongly inhibited p-PRAS40 across all cell lines and an increase in p-AKT was observed at both Ser473 and Thr308, which has been previously described (29). Pathway suppression was observed with either PI3Kβ/δi or PI3Kα/δi but did not achieve similar levels of inhibition as with AKTi or mTORC1/2i. In the PI3Kβ/δi/PI3Kα/δi sensitive cell lines there was not a clear pattern of pathway suppression to differentiate from the cell lines resistant to the PI3K inhibitors. Suppression of p-S6RP was observed in the *PIK3CA* mutant cell line following PI3Kα/δi inhibition. We also determined MYC protein levels following inhibitor treatment and observed reduced levels of MYC after 24 hours AKTi treatment in all T-ALL cell lines. PI3K and mTOR inhibition did not supress MYC protein levels to the same extent as AKTi, with no clear pattern of MYC suppression observed. In summary, we find that AKT and mTOR inhibition consistently suppressed S6RP phosphorylation, which correlates with sensitivity in the T-ALL cell lines.

### Differential activation of PI3K pathway signaling in T-ALL

**Figure 4:**
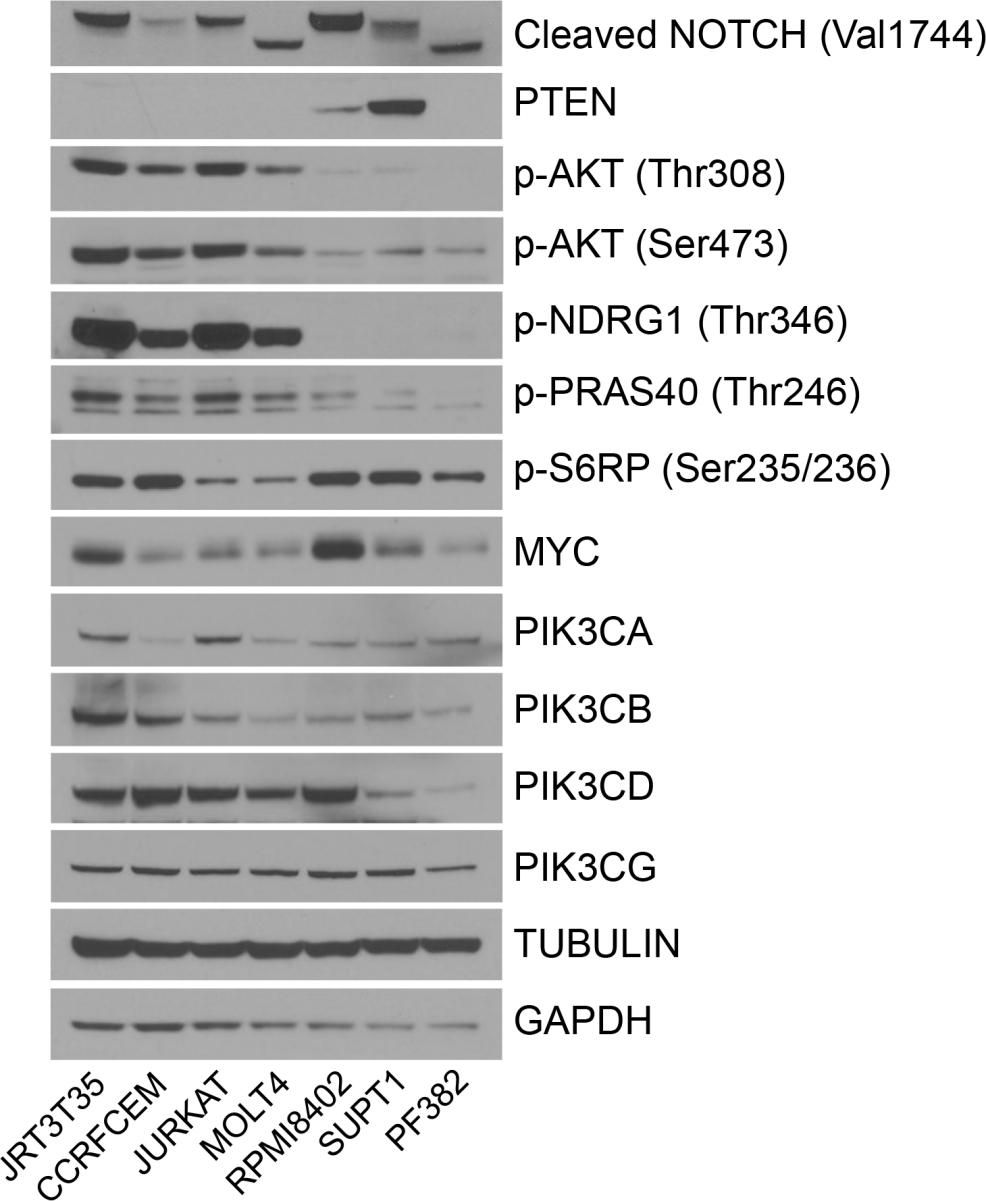
Basal levels of protein expression and activation in the T-ALL cell lines Untreated protein lysate was extracted from seven T-ALL cell lines and Western blot analysis performed to determine the relative expression of the indicated proteins and phosphorylated proteins (*n* = 2).

In addition to examining the regulation of pathway biomarkers, we also determined the basal levels of protein expression and activation across the T-ALL cell lines. *NOTCH* is frequently mutated in T-ALL and genetics data for the seven cell lines suggest that *NOTCH* is mutated in all of these cell lines [5, 23]. In accordance with this, the cleaved form of NOTCH, which is indicative of active NOTCH signaling, was observed in all seven T-ALL cell lines (Figure 4). Five out of seven cell lines also have deletion of *PTEN*, which is observed at the protein level. Ser473 and Thr308 AKT phosphorylation was elevated in four of the T-ALL cell lines. In the same four cell lines, there was also strong phosphorylation of NDRG1 compared with the other three cell lines. p-PRAS40 was also elevated in the same four cell lines but not to the same extent as for p-NDRG1 and p-AKT. MYC protein levels were variable across the cell lines, and no correlation could be made between PI3K isoform expression and PI3K inhibitor sensitivity. These results demonstrate that a subset of NOTCH mutant T-ALL cell lines can be grouped through their protein expression profile as being PTEN null and p-AKT/p-NDRG1 high.

### PTEN loss and active AKT/NDRG1 predicts AZD5363-induced apoptotic response

**Figure 5:**
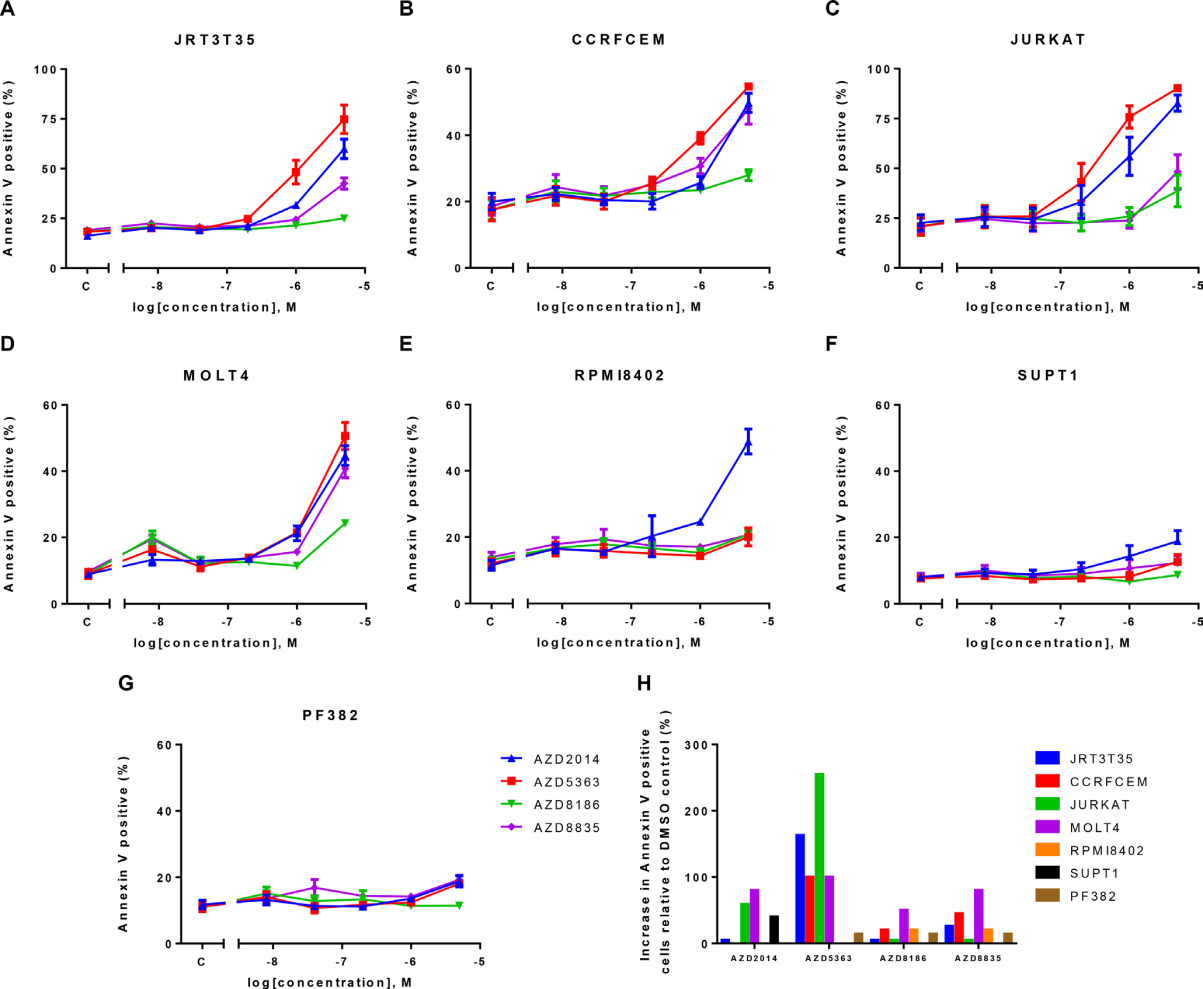
AKT inhibition induces apoptosis in four out of seven T-ALL cell lines A panel of T-ALL cell lines were treated with a dose response of four PI3K pathway inhibitors. Annexin V flow cytometry was performed 72 hours post inhibitor treatment. (**A–G**) mean ± S.E.M. percentage of Annexin V positive cells (*n* = 2). (**H**) Mean percentage increase of Annexin V positive cells relative to DMSO control at the following drug concentrations (AZD2014–0.25 μM, AZD5363–1 μM, AZD8186-0.5 μM and AZD8835-0.5 μM). S.E.M = standard error of the mean.

Given the strong phenotype following AKT or mTOR inhibition, we wanted to determine whether these inhibitors induced apoptosis in the T-ALL cell lines. Cells were dosed with increasing concentrations of each compound and Annexin V flow cytometry was performed 72 hours post drug treatment. At the concentrations defined in the Western blot experiments, AKTi induced apoptosis in four out of seven cell lines, whereas we did not observe an increase in apoptosis following PI3K or mTOR inhibitor treatment (Figure 5). At higher drug concentrations we did observe apoptosis with the PI3K pathway inhibitors. Interestingly the four cell lines that undergo AKTi-induced apoptosis are the same cell lines that we previously described as *PTEN* null/p-AKT/p-NDRG1 high. These results suggest that at relevant concentrations of the inhibitors, only AKTi induces apoptosis and this is in a defined subset of the T-ALL cell lines.

## DISCUSSION

Here we screened four PI3K pathway inhibitors, targeting PI3Kα/δ, PI3Kβ/δ, AKT and mTORC1/2, against a panel of 971 cancer cell lines. Using elastic net regularization, we confirmed the robustness of the cell panel screen and observed a number of sensitivity correlations that have been previously described. Whilst we focussed on hematological malignancies and T-ALL, this PI3K pharmacology dataset can be used as a resource for further interrogation and validation of PI3K pathway biology and sensitivity. In addition to T-ALL, there were a number of other hematological subtypes that displayed enhanced sensitivity to the PI3K pathway inhibitors, which are currently being investigated.

Given that five out of seven cell lines are *PTEN* null, we did not observe strong sensitivity to the PI3K inhibitors across the T-ALL cell line panel. Previous reports have described that the PI3Kδ and PI3Kγ isoforms are required for the development of *PTEN* null T-ALL [30–32]. Further investigation is required to determine whether *NOTCH* mutations negate the PI3Kδ/γ dependency in the T-ALL cell lines. PI3K isoform selectivity of the PI3K-selective inhibitors was determined in enzyme and cell based assays and demonstrate that GDC0941 and BYL719 inhibit the PI3Kδ and PI3Kγ isoforms at sub-micromolar concentrations (Supplementary Table 4) [20]. GDC0941 was active in three out of seven cell lines, which suggests that there may be alternative mechanisms that lead to the T-ALL AKT dependency. Although a number of the PI3K inhibitors screened inhibit the PI3Kγ isoform, an isoform-selective PI3Kγ inhibitor would help discriminate the relative contribution of the different PI3K isoforms.

High PI3K activity, as determined by phosphorylated AKT did not correlate with sensitivity to the PI3K inhibitors, although all of the cell lines were sensitive to AKT inhibition. This implies that there are other signaling events that confer AKT dependency in T-ALL. Our findings are in concordance with other reports that demonstrate a dependence on AKT and mTOR signaling in T-ALL, but this study suggests that targeting AKT or mTOR in *NOTCH*-mutant T-ALL would be advantageous over PI3K inhibitors [33–37].

An overlapping feature of AKT and mTOR inhibition was the ability to suppress S6RP phosphorylation in the T-ALL cell lines. These results suggests that the targeting of S6RP may be critical to induce the phenotype, and inhibition of S6RP has been previously linked to efficacy [38]. In the Western blot experiments, two out of three cell lines that are sensitive to the PI3K inhibitors were treated with a concentration of PI3Kβ/δi or PI3Kα/δi (0.5 μM) that was below their IC_50_ value for growth inhibition and maintained their PI3K isoform selectivity. Determining S6RP phosphorylation at a concentration above the IC_50_ of T-ALL cell lines sensitive to PI3Kβ/δi and PI3Kα/δi will strengthen the S6RP correlation to the phenotype.

In addition to the inhibition of cell proliferation observed for AKT and mTOR inhibitors, we also demonstrated apoptosis in four out of seven T-ALL cell lines following AKT but not mTOR inhibition. These results may provide a rationale for targeting AKT rather than mTOR, and may reflect the function of many AKT substrates as inhibitors of apoptosis [39, 40]. Additional studies are required to determine if the AKT inhibitors enhance efficacy *in vivo* over the mTOR compounds. In conclusion, we have revealed a dependence of blood/lymph lineage cell lines to PI3K pathway signaling. Further investigation of the role of the PI3K pathway signaling in T-ALL revealed novel dependencies of the different signaling nodes, with AKT and mTOR critical for the survival of *NOTCH*-mutant T-ALL cell lines. The results merit further investigation for AKT and mTOR inhibitors as potential treatments for T-ALL.

## MATERIALS AND METHODS

### Cells and cell culture

SUPT1 and MOLT4 cells were purchased from ATCC. JURKAT, JRT3T35, PF382 and RPMI8402 cells were purchased from DSMZ. CCRFCEM were obtained from the Japanese Collection of Research Bioresources Cell Bank. Cells lines were grown in RPMI-1640 buffer + 10% FCS + 2 mM glutamine at 37°C, 5% carbon dioxide. All cell lines were authenticated at AstraZeneca cell banking using DNA fingerprinting short tandem repeat assays. All revived cells were used within 15 passages, and cultured for less than 6 months.

### Reagents and antibodies

All inhibitors were dissolved in DMSO to a concentration of 10 mM and stored under nitrogen. The following antibodies were purchased from Cell Signaling Technology (Danvers, MA, USA): Cleaved Notch1 (#4147), PTEN (#9559), pAKT T308 (#2965), pAKT S473 (#9271), pNDRG1 T346 (#5482), pS6RP S235/236 (#2211), PIK3CA (#4249), PIK3CB (#3011), PIK3CG (#5405), TUBULIN (#5346), GAPDH (#3683). PIK3CD (sc-7176), pPRAS40 T246 (44-1100G) and MYC (ab32072) were purchased from Santa Cruz (Dallas, TX, USA), Life Technologies (Grand Island, NY, USA) and Abcam (Cambridge, UK), respectively.

### Screening assays

The Sanger cancer cell line pharmacology screen was performed as previously described [41]. For the cell proliferation validation screen, the experiment was set up as previously defined [16]. Pre-dose measurements were made and IC_50_ values were determined using live cell counts. Formulas used to calculate growth curves and to analyse combination data have been previously described [28]. Briefly, combination activity (synergism) was analyzed in Genedata Screener12 (Genedata, Basel, Switzerland) using the Loewe dose-additivity model. This model of additivity provides a null reference that is predicted by the expected response if the two agents were the same drug. The 3-dimensional model surface, predicted from the two single-agent response curves, is subtracted from the experimentally-derived 3-dimensional dose effect surface to generate a difference volume. This excess matrix volume can be integrated to generate a synergy score. A synergy score cut-off > 5 would be used to identify combinations of interest.

### Protein extraction and western blotting

Cells were lysed in RIPA buffer (Life Technologies) supplemented with 1× Protease Inhibitor Cocktail (Roche, Welwyn Garden City, UK), 1× phosphatase inhibitor (Life Technologies) and 1:5,000 benzonase (Sigma, Gillingham, UK) and equal amounts of protein were loaded and separated by SDS-PAGE. Horseradish peroxidase-linked secondary antibodies (GE Healthcare, Little Chalfont, UK) and ECL or supersignal (Life Technologies) were used to detect immune complexes.

### Flow cytometry

The PE Annexin V apoptosis detection kit (BD Biosciences, Oxford, UK) was used for flow cytometry, performed using a Guava easyCyte flow cytometer (MERCK Millipore, Watford, UK).

### Bioinformatic analysis

For the elastic net regression analysis, we used as input variables to the elastic net algorithm the natural log IC_50_ responses for each of the 971 cell lines together with a matrix of genomic features (gene mutations and lineage) represented as binary values for each of 1758 cancer-relevant genes and 39 cancer lineages. Strongly damaging loss-of-function mutations comprising nonsense, frameshift and splice site mutations in 189 well-characterised tumor suppressor genes were manually curated and classified into binary values. Prediction performance was determined using tenfold cross-validation and the elastic net features were validated to retain only those that were consistent across runs. The glmnet 1.9-5 software package and R 3.0.1 were used to perform the elastic net regression analysis [42, 43].

## SUPPLEMENTARY MATERIALS FIGURES AND TABLES



## References

[1] Van Vlierberghe P, Ferrando A (2012). The molecular basis of T cell acute lymphoblastic leukemia. The Journal of Clinical Investigation.

[2] Blackburn JS, Liu S, Raiser DM, Martinez SA, Feng H, Meeker ND, Gentry J, Neuberg D, Look AT, Ramaswamy S, Bernards A, Trede NS, Langenau DM (2012). Notch signaling expands a pre-malignant pool of T-cell acute lymphoblastic leukemia clones without affecting leukemia-propagating cell frequency. Leukemia.

[3] Gutierrez A, Sanda T, Grebliunaite R, Carracedo A, Salmena L, Ahn Y, Dahlberg S, Neuberg D, Moreau LA, Winter SS, Larson R, Zhang J, Protopopov A (2009). High frequency of PTEN, PI3K, and AKT abnormalities in T-cell acute lymphoblastic leukemia. Blood.

[4] Roti G, Stegmaier K (2014). New Approaches to Target T-ALL. Frontiers in Oncology.

[5] Weng AP, Ferrando AA, Lee W, Morris JP, Silverman LB, Sanchez-Irizarry C, Blacklow SC, Look AT, Aster JC (2004). Activating Mutations of NOTCH1 in Human T Cell Acute Lymphoblastic Leukemia. Science.

[6] Martelli AM, Evangelisti C, Chappell W, Abrams SL, Basecke J, Stivala F, Donia M, Fagone P, Nicoletti F, Libra M, Ruvolo V, Ruvolo P, Kempf CR (2011). Targeting the translational apparatus to improve leukemia therapy: roles of the PI3K/PTEN/Akt/mTOR pathway. Leukemia.

[7] Cully M, You H, Levine AJ, Mak TW (2006). Beyond PTEN mutations: the PI3K pathway as an integrator of multiple inputs during tumorigenesis. Nat Rev Cancer.

[8] Kofuji S, Kimura H, Nakanishi H, Nanjo H, Takasuga S, Liu H, Eguchi S, Nakamura R, Itoh R, Ueno N, Asanuma K, Huang M, Koizumi A (2015). INPP4B Is a PtdIns (3, 4, 5) P3 Phosphatase That Can Act as a Tumor Suppressor. Cancer Discovery.

[9] Laplante M, Sabatini DM (2009). mTOR signaling at a glance. Journal of Cell Science.

[10] Liu P, Cheng H, Roberts TM, Zhao JJ (2009). Targeting the phosphoinositide 3-kinase pathway in cancer. Nat Rev Drug Discov.

[11] Vanhaesebroeck B, Stephens L, Hawkins P (2012). PI3K signalling: the path to discovery and understanding. Nat Rev Mol Cell Biol.

[12] Yuan TL, Cantley LC (2008). PI3K pathway alterations in cancer: variations on a theme. Oncogene.

[13] Hancox U, Cosulich S, Hanson L, Trigwell C, Lenaghan C, Ellston R, Dry H, Crafter C, Barlaam B, Fitzek M, Smith PD, Ogilvie D, D'Cruz C (2015). Inhibition of PI3Kβ Signaling with AZD8186 Inhibits Growth of PTEN-Deficient Breast and Prostate Tumors Alone and in Combination with Docetaxel. Molecular Cancer Therapeutics.

[14] Jia S, Liu Z, Zhang S, Liu P, Zhang L, Lee SH, Zhang J, Signoretti S, Loda M, Roberts TM, Zhao JJ (2008). Essential roles of PI(3)K-p110β in cell growth, metabolism and tumorigenesis. Nature.

[15] Wee S, Wiederschain D, Maira S-M, Loo A, Miller C, deBeaumont R, Stegmeier F, Yao YM, Lengauer C (2008). PTEN-deficient cancers depend on PIK3CB. Proceedings of the National Academy of Sciences.

[16] Davies BR, Greenwood H, Dudley P, Crafter C, Yu D-H, Zhang J, Li J, Gao B, Ji Q, Maynard J, Ricketts SA, Cross D, Cosulich S (2012). Preclinical Pharmacology of AZD5363, an Inhibitor of AKT: Pharmacodynamics, Antitumor Activity, and Correlation of Monotherapy Activity with Genetic Background. Molecular Cancer Therapeutics.

[17] Hofmann C, Stühmer T, Schmiedl N, Wetzker R, Mottok A, Rosenwald A, Langer C, Zovko J, Chatterjee M, Einsele H, Bargou RC, Steinbrunn T (2014). PI3K-dependent multiple myeloma cell survival is mediated by the PIK3CA isoform. British Journal of Haematology.

[18] Yang W, Soares J, Greninger P, Edelman EJ, Lightfoot H, Forbes S, Bindal N, Beare D, Smith JA, Thompson IR, Ramaswamy S, Futreal PA, Haber DA (2013). Genomics of Drug Sensitivity in Cancer (GDSC): a resource for therapeutic biomarker discovery in cancer cells. Nucleic Acids Res.

[19] Guichard SM, Curwen J, Bihani T, D'Cruz CM, Yates JW, Grondine M, Howard Z, Davies B, Bigley G, Klinowska T, Pike KG, Pass M, Chresta CM (2015). AZD2014, an inhibitor of mTORC1 and mTORC2, is highly effective in ER+ breast cancer when administered using intermittent or continuous schedules. Molecular Cancer Therapeutics.

[20] Hudson K, Hancox U, Trigwell C, McEwen R, Polanska U, Nikolaou M, Gutierrez PM, Avivar-Valderas A, Delpuech O, Dudley P, Hanson L, Ellston R, Jones A (2016). Intermittent high dose scheduling of AZD8835, a novel selective inhibitor of PI3Kα and PI3Kδ, demonstrates treatment strategies for PIK3CA-dependent breast cancers. Molecular Cancer Therapeutics.

[21] Zou H, Hastie T (2005). Regularization and variable selection via the elastic net. Journal of the Royal Statistical Society: Series B (Statistical Methodology).

[22] Younes A, Samad N (2011). Utility of mTOR Inhibition in Hematologic Malignancies. The Oncologist.

[23] Forbes SA, Beare D, Gunasekaran P, Leung K, Bindal N, Boutselakis H, Ding M, Bamford S, Cole C, Ward S, Kok CY, Jia M, De T (2015). COSMIC: exploring the world's knowledge of somatic mutations in human cancer. Nucleic Acids Res.

[24] Folkes AJ AK, Alderton WK, Alix S, Baker SJ, Box G, Chuckowree IS, Clarke PA, Depledge P, Eccles SA, Friedman LS, Hayes A, Hancox TC (2008). The identification of 2-(1H-indazol-4-yl)-6-(4-methanesulfonyl-piperazin-1-ylmethyl)-4-morpholin-4-yl-thieno [3, 2-d]pyrimidine (GDC-0941) as a potent, selective, orally bioavailable inhibitor of class I PI3 kinase for the treatment of cancer. Journal of Medicinal Chemistry.

[25] Furet P, Guagnano V, Fairhurst RA, Imbach-Weese P, Bruce I, Knapp M, Fritsch C, Blasco F, Blanz J, Aichholz R, Harmon J, Fabbro D, Caravatti G (2013). Discovery of NVP-BYL719 a potent and selective phosphatidylinositol-3 kinase alpha inhibitor selected for clinical evaluation. Bioorganic & Medicinal Chemistry Letters.

[26] Ikeda H, Hideshima T, Fulciniti M, Perrone G, Miura N, Yasui H, Okawa Y, Kiziltepe T, Santo L, Vallet S, Cristea D, Calabrese E, Gorgun G (2010). PI3K/p110δ is a novel therapeutic target in multiple myeloma. Blood.

[27] Schwartz S, Wongvipat J, Trigwell Cath B, Hancox U, Carver Brett S, Rodrik-Outmezguine V, Will M, Yellen P, de Stanchina E, Baselga J, Scher HI, Barry ST, Sawyers CL (2015). Feedback Suppression of PI3Kα Signaling in PTEN-Mutated Tumors Is Relieved by Selective Inhibition of PI3Kβ. Cancer Cell.

[28] Crafter C, Vincent JP, Tang E, Dudley P, James NH, Klinowska T, Davies BR (2015). Combining AZD8931, a novel EGFR/HER2/HER3 signalling inhibitor, with AZD5363 limits AKT inhibitor induced feedback and enhances antitumour efficacy in HER2-amplified breast cancer models. International Journal of Oncology.

[29] Okuzumi T, Fiedler D, Zhang C, Gray DC, Aizenstein B, Hoffman R, Shokat KM (2009). Inhibitor hijacking of Akt activation. Nat Chem Biol.

[30] Lonetti A, Antunes IL, Chiarini F, Orsini E, Buontempo F, Ricci F, Tazzari PL, Pagliaro P, Melchionda F, Pession A, Bertaina A, Locatelli F, McCubrey JA (2014). Activity of the pan-class I phosphoinositide 3-kinase inhibitor NVP-BKM120 in T-cell acute lymphoblastic leukemia. Leukemia.

[31] Spijkers-Hagelstein JAP, Pinhancos SS, Schneider P, Pieters R, Stam RW (2014). Chemical genomic screening identifies LY294002 as a modulator of glucocorticoid resistance in MLL-rearranged infant ALL. Leukemia.

[32] Subramaniam Prem S, Whye Dosh W, Efimenko E, Chen J, Tosello V, De Keersmaecker K, Thompson MA, Castillo M, Cordon-Cardo C, Dave UP, Ferrando A, Lannutti BJ (2012). Targeting Nonclassical Oncogenes for Therapy in T-ALL. Cancer Cell.

[33] Chan SM, Weng AP, Tibshirani R, Aster JC, Utz PJ (2007). Notch signals positively regulate activity of the mTOR pathway in T-cell acute lymphoblastic leukemia. Blood.

[34] Evangelisti C, Ricci F, Tazzari P, Tabellini G, Battistelli M, Falcieri E, Chiarini F, Bortul R, Melchionda F, Pagliaro P, Pession A, McCubrey JA, Martelli AM (2011). Targeted inhibition of mTORC1 and mTORC2 by active-site mTOR inhibitors has cytotoxic effects in T-cell acute lymphoblastic leukemia. Leukemia.

[35] Gu L, Zhou C, Liu H, Gao J, Li Q, Mu D, Ma Z (2010). Rapamycin sensitizes T-ALL cells to dexamethasone-induced apoptosis. Journal of Experimental & Clinical Cancer Research.

[36] Levy DS, Kahana JA, Kumar R (2009). AKT inhibitor, GSK690693, induces growth inhibition and apoptosis in acute lymphoblastic leukemia cell lines. Blood.

[37] Simioni C, Neri LM, Tabellini G, Ricci F, Bressanin D, Chiarini F, Evangelisti C, Cani A, Tazzari PL, Melchionda F, Pagliaro P, Pession A, McCubrey JA (2012). Cytotoxic activity of the novel Akt inhibitor, MK-2206, in T-cell acute lymphoblastic leukemia. Leukemia.

[38] Elkabets M, Vora S, Juric D, Morse N, Mino-Kenudson M, Muranen T, Tao J, Campos AB, Rodon J, Ibrahim YH, Serra V, Rodrik-Outmezguine V, Hazra S (2013). mTORC1 Inhibition Is Required for Sensitivity to PI3K p110α Inhibitors in PIK3CA-Mutant Breast Cancer. Science Translational Medicine.

[39] Altomare DA, Testa JR (2005). Perturbations of the AKT signaling pathway in human cancer. Oncogene.

[40] Reynolds C, Roderick JE, LaBelle JL, Bird G, Mathieu R, Bodaar K, Colon D, Pyati U, Stevenson KE, Qi J, Harris M, Silverman LB, Sallan SE (2014). Repression of BIM mediates survival signaling by MYC and AKT in high-risk T-cell acute lymphoblastic leukemia. Leukemia.

[41] Garnett MJ, Edelman EJ, Heidorn SJ, Greenman CD, Dastur A, Lau KW, Greninger P, Thompson IR, Luo X, Soares J, Liu Q, Iorio F, Surdez D (2012). Systematic identification of genomic markers of drug sensitivity in cancer cells. Nature.

[42] Friedman J, Hastie T, Tibshirani R (2010). Regularization Paths for Generalized Linear Models via Coordinate Descent. Journal of statistical software.

[43] R Development Core Team (2015). R: A Language and Environment for Statistical Computing. R Foundation for Statistical Computing.

