# Supplementary material for: Identification of differential PI3K pathway target dependencies in T-cell acute lymphoblastic leukemia through a large cancer cell panel screen
Source: Oncotarget. 2016 Mar 10;7(16):22128–39. doi: 10.18632/oncotarget.8031 (PMC5008349; doi:10.18632/oncotarget.8031)
Supplement: Supplementary file 1 [file oncotarget-07-22128-s001.pdf]

Identification of differential PI3K pathway target dependencies in T-cell acute lymphoblastic leukemia through a large cancer cell panel screen

Supplementary Materials

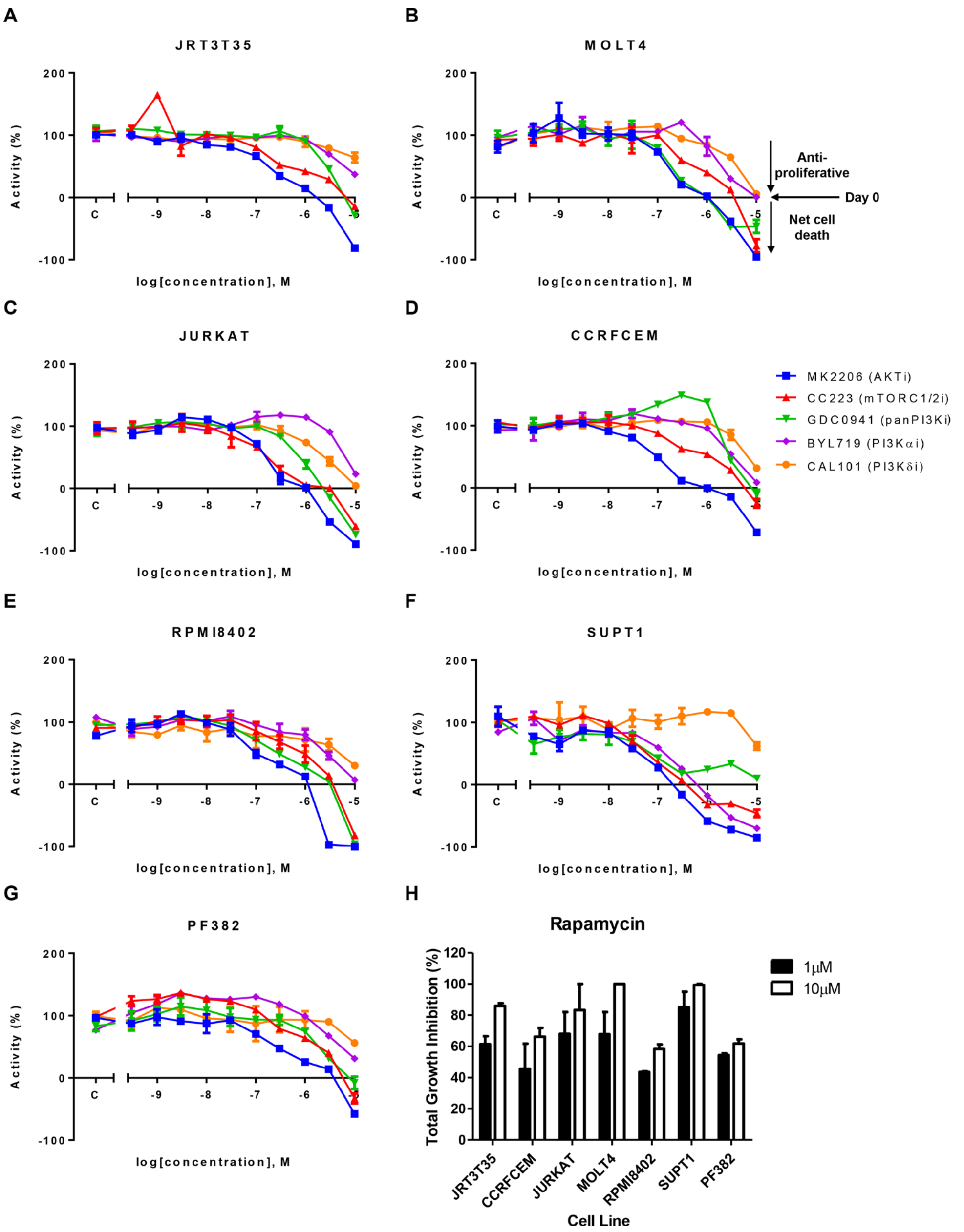

Supplementary Figure S1: A screen targeting multiple nodes of the PI3K pathway identify their differential requirements in T-ALL. A panel of T-ALL cell lines were treated with a dose response of different PI3K pathway inhibitors. Live cell number was assessed after five days using a sytox green endpoint. (**A–G**) Exemplar mean ± S.E.M. dose response curves for the inhibitors across the T-ALL cell line panel. Zero activity represents the initial seeding density on the day of dosing (Day 0) ($n = 2$). (h) mean ± S.E.M. growth inhibition following rapamycin treatment across the cell line panel ($n = 2$). S.E.M = standard error of the mean.

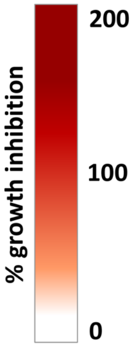

Row 1: AZD2014 [μM] vs AZD8835 [μM] (Synergy Score 0.744)

Fitted Data

AZD2014 [μM] \ AZD8835 [μM]	0	1E-3	3.571E-3	0.011	0.032	0.1	0.357	1.071
1.071	113	117	112	112	113	113	114	111
0.357	108	107	108	108	108	109	112	109
0.1	92	88	94	92	92	94	102	101
0.032	45	47	50	51	60	69	97	114
0.011	27	30	26	24	30	36	73	110
3.571E-3	9	14	12	9	12	17	54	106
1E-3	0	4	6	3	2	6	37	105
0	-9	-13	-2	-4	-8	1	36	103

Model Loewe

AZD2014 [μM] \ AZD8835 [μM]	0	1E-3	3.571E-3	0.011	0.032	0.1	0.357	1.071
1.071	112	112	112	112	113	113	113	114
0.357	107	107	107	107	107	108	109	113
0.1	90	90	90	90	91	92	97	109
0.032	61	61	61	61	62	66	79	104
0.011	29	29	29	30	32	39	62	100
3.571E-3	8	8	8	9	11	20	51	99
1E-3	-3	-3	-3	-2	0	9	45	98
0		-11	-10	-10	-7	3	43	98

Excess Loewe

AZD2014 [μM] \ AZD8835 [μM]	0	1E-3	3.571E-3	0.011	0.032	0.1	0.357	1.071
1.071	0	4	-1	0	1	1	1	-4
0.357	1	0	1	1	1	2	3	-4
0.1	2	-2	4	2	1	2	5	-7
0.032	-15	-14	-10	-10	-2	3	18	11
0.011	-2	1	-3	-6	-2	-3	12	10
3.571E-3	1	7	4	0	1	-2	4	7
1E-3	3	7	9	5	2	-3	-8	7
0		-3	9	6	0	-2	-6	5

Synergy Score
0.744

Row 2: AZD8835 [μM] vs AZD8186 [μM] (Synergy Score -2.1)

Fitted Data

AZD8835 [μM] \ AZD8186 [μM]	0	1E-3	3.571E-3	0.011	0.032	0.1	0.357	1.071
1.071	101	99	98	100	97	99	98	82
0.357	63	55	61	58	60	56	58	61
0.1	27	24	29	24	29	25	34	59
0.032	17	15	17	14	17	17	44	65
0.011	12	11	10	9	13	15	29	57
3.571E-3	14	12	11	10	11	13	22	57
1E-3	11	10	8	7	8	11	23	59
0	15	13	11	11	13	18	33	64

Model Loewe

AZD8835 [μM] \ AZD8186 [μM]	0	1E-3	3.571E-3	0.011	0.032	0.1	0.357	1.071
1.071	102	102	102	102	102	102	103	106
0.357	67	67	67	67	68	70	77	89
0.1	27	27	27	28	30	36	51	73
0.032	17	17	17	18	21	27	43	67
0.011	15	15	16	17	19	25	41	66
3.571E-3	15	15	15	16	19	24	40	65
1E-3	15	15	15	16	18	24	40	65
0		15	15	16	18	24	40	65

Excess Loewe

AZD8835 [μM] \ AZD8186 [μM]	0	1E-3	3.571E-3	0.011	0.032	0.1	0.357	1.071
1.071	-1	-2	-3	-2	-5	-3	-5	-24
0.357	-4	-12	-6	-10	-8	-15	-19	-27
0.1	0	-3	2	-4	-1	-11	-16	-14
0.032	0	-1	-1	-5	-4	-10	1	-2
0.011	-3	-4	-5	-7	-7	-10	-12	-9
3.571E-3	-1	-3	-5	-7	-8	-12	-18	-8
1E-3	-4	-5	-8	-9	-10	-13	-16	-5
0		-2	-4	-5	-5	-6	-6	0

-2.1

Row 3: AZD2014 [μM] vs AZD8186 [μM] (Synergy Score -0.3)

Fitted Data

AZD2014 [μM] \ AZD8186 [μM]	0	1E-3	3.571E-3	0.011	0.032	0.1	0.357	1.071
1.071	114	113	115	113	113	113	114	114
0.357	108	107	107	108	107	107	109	110
0.1	91	88	88	91	90	90	94	97
0.032	66	64	63	64	67	66	71	81
0.011	31	30	30	28	35	32	45	81
3.571E-3	12	14	12	12	17	17	34	65
1E-3	2	6	3	4	3	3	19	54
0	-10	-5	-10	-6	-13	-7	15	42

Model Loewe

AZD2014 [μM] \ AZD8186 [μM]	0	1E-3	3.571E-3	0.011	0.032	0.1	0.357	1.071
1.071	115	115	115	115	115	115	115	116
0.357	108	108	108	108	108	109	109	111
0.1	91	91	91	91	91	92	94	99
0.032	64	64	64	64	65	66	71	84
0.011	34	34	34	34	35	38	47	69
3.571E-3	12	12	12	13	13	17	29	60
1E-3	-1	-1	-1	0	1	4	18	55
0		-11	-11	-11	-10	-6	13	53

Excess Loewe

AZD2014 [μM] \ AZD8186 [μM]	0	1E-3	3.571E-3	0.011	0.032	0.1	0.357	1.071
1.071	-1	-2	0	-1	-1	-2	-1	-2
0.357	0	-1	-1	0	-2	-1	0	-1
0.1	0	-3	-4	-1	-2	-2	0	-2
0.032	2	0	-1	0	3	0	0	-2
0.011	-3	-4	-4	-6	-1	-5	-2	12
3.571E-3	0	1	0	-1	4	1	5	5
1E-3	3	6	3	4	3	0	1	-2
0		6	1	5	-3	-1	2	-12

-0.3

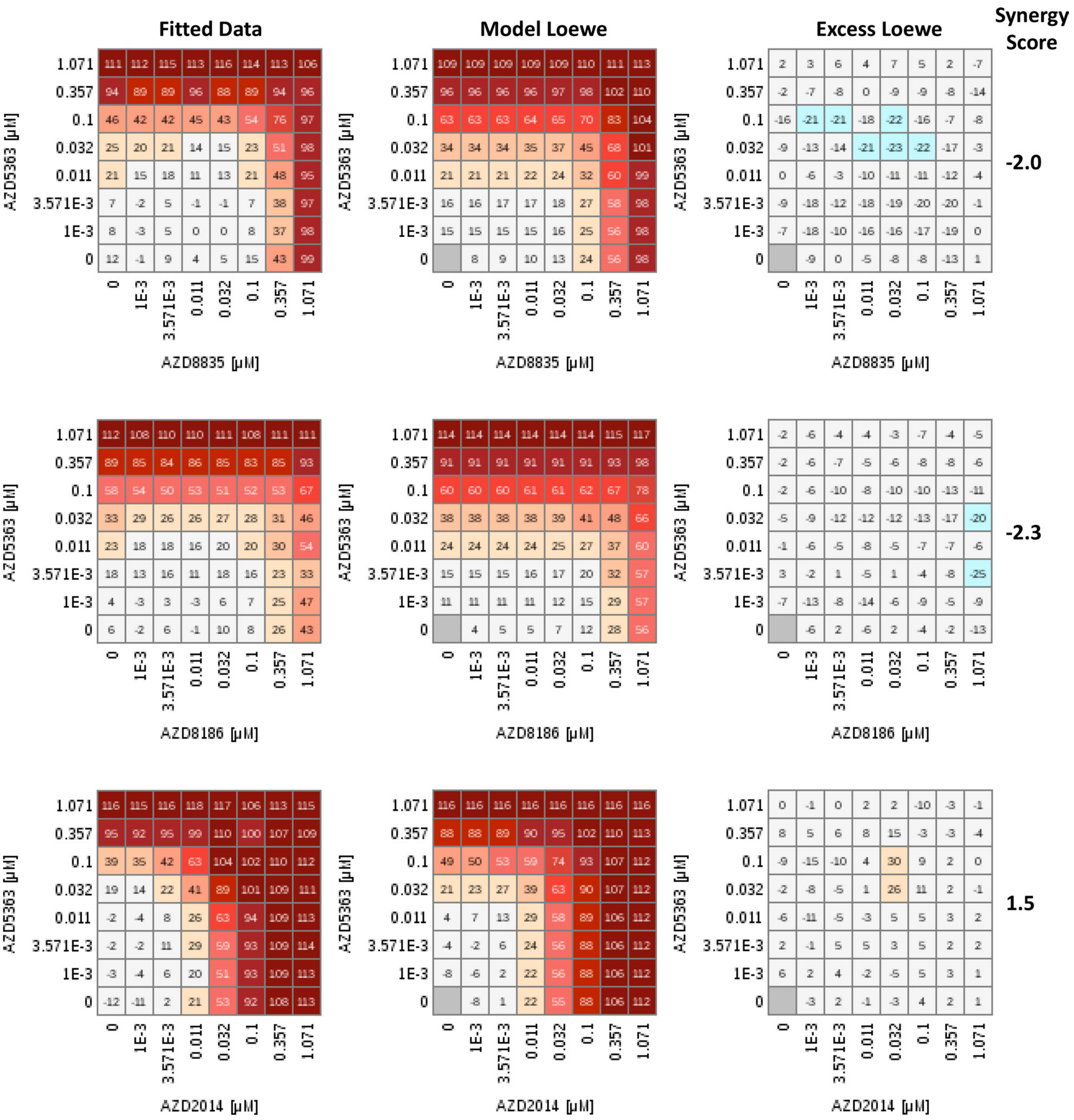

Supplementary Figure S2: PI3K intra-pathway combinations induce additive but not synergistic effects in T-ALL. A panel of T-ALL cell lines were treated with a dose response of different PI3K pathway inhibitors in a 8 × 8 dose response matrix. Live cell number was assessed after five days using a sytox green endpoint and synergy scores were generated using the Loewe model of additivity. Representative dose matrices and synergy scores from one experiment from the SUPT1 cell line. Fitted data: dose matrix representing percent growth inhibition values. Model Loewe: Loewe model of additivity calculated from the monotherapy dose response curves. Excess Loewe: Excess heatmap (synergy) calculated by subtracting the Loewe model of additivity data from the fitted data. Calculated synergy scores for each cell line are shown in the far right column.

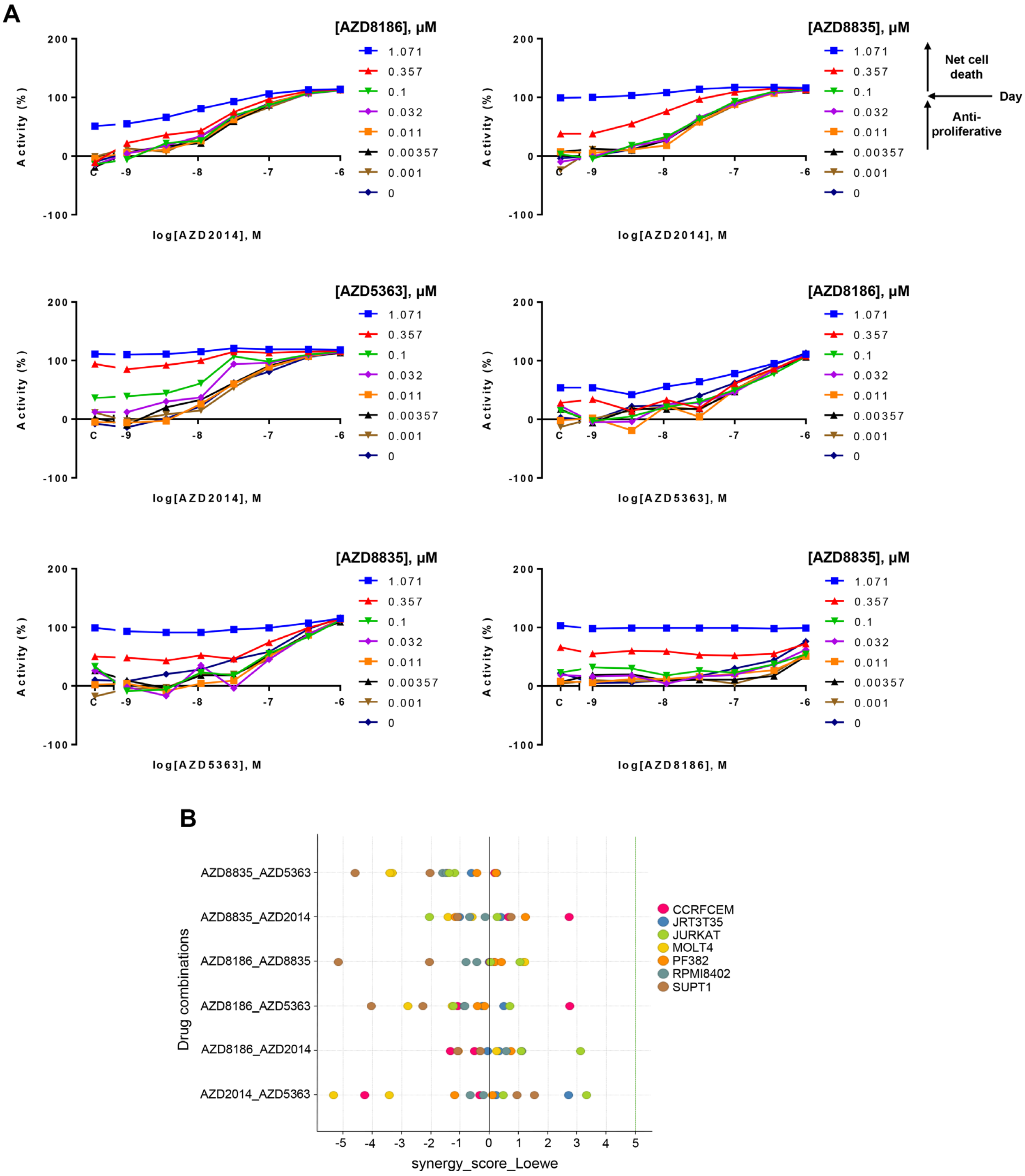

Supplementary Figure S3: PI3K intra-pathway combinations induce additive but not synergistic effects in T-ALL. A panel of T-ALL cell lines were treated with a dose response of different PI3K pathway inhibitors in a 8 × 8 dose response matrix. Live cell number was assessed after 5 days using a sytox green endpoint and synergy scores were generated using the Loewe model of additivity. (**A**) Exemplar mean dose response curves for the inhibitor combinations in the SUPT1 cell line. 100% activity represents the initial seeding density on the day of dosing (Day 0) ($n = 2$). (**B**) Comparison of mean synergy scores for the PI3K intra-pathway combinations across the T-ALL cell lines ($n = 2$). S.E.M = standard error of the mean.

Supplementary Table 1: Sanger cancer cell line pharmacology results

Cell Line	Tissue	AZD2014 (IC_{50} uM)	AZD5363 (IC_{50} uM)	AZD8186 (IC_{50} uM)	AZD8835 (IC_{50} uM)
HUCCT1	biliary tract	0.248	72.199	2.911	65.066
EGI1	biliary tract	0.447	429.161	9.006	11.756
ETK1	biliary tract	0.478	43.247	8.249	5.836
TGBC1TKB	biliary tract	0.684	34.178	15.719	315.880
TGBC24TKB	biliary tract	0.761	24.288	68.985	3.673
VMCUB1	bladder	0.119	137.852	7.460	72.429
CAL29	bladder	0.187	1.552	4.628	0.658
SW780	bladder	0.190	47.688	4.924	152.344
647V	bladder	0.254	64.434	2.735	49.061
RT112	bladder	0.267	8.427	8.595	7.475
TCCSUP	bladder	0.275	26.510	11.813	8.067
UMUC3	bladder	0.397	40.195	3.750	29.481
BFTC905	bladder	0.397	164.294	6.309	2.358
SW1710	bladder	0.519	2.631	0.997	8.654
SCABER	bladder	0.552	12.908	5.458	43.158
KU1919	bladder	0.722	35.541	100.822	126.278
5637	bladder	0.726	45.177	8.552	41.184
J82	bladder	0.819	39.122	11.059	42.789
HT1197	bladder	1.074	43.599	14.050	3.673
T24	bladder	1.181	47.341	3.438	26.019
LB831BLC	bladder	1.560	16.495	23.080	13.142
HT1376	bladder	3.550	148.414	10.564	53.431
DSH1	bladder	3.565	1.704	327.278	31.022
RT4	bladder	3.791	3443.195	79.547	43.591
HH	blood/lymph	0.015	15.159	0.075	0.243
CCRFCEM	blood/lymph	0.038	0.231	13.779	6.943
SUDHL5	blood/lymph	0.038	0.300	0.036	0.187
CROAP3	blood/lymph	0.041	7.219	45.969	68.326
H929	blood/lymph	0.043	0.003	0.729	0.285
RAMOS2G64C10	blood/lymph	0.044	1.517	14.669	287.287
GA10	blood/lymph	0.046	5.785	3.171	13.316
MOLT4	blood/lymph	0.046	0.277	0.530	0.811
SUPM2	blood/lymph	0.047	110.527	2.201	3.174
MHHPREB1	blood/lymph	0.049	6.218	1.450	6.082
P30OHK	blood/lymph	0.049	8.710	0.287	0.222
MOLM16	blood/lymph	0.050	1.094	0.341	0.989
L363	blood/lymph	0.051	0.273	1.993	0.525
GRST	blood/lymph	0.052	1.208	3.481	1.874
EM2	blood/lymph	0.055	2.082	8.070	8.349
MEC1	blood/lymph	0.062	5.479	0.436	0.320
697	blood/lymph	0.069	5.329	1.991	1.396
RPMI8402	blood/lymph	0.075	3.659	0.218	1.100
KCL22	blood/lymph	0.076		28.319	36.555
TUR	blood/lymph	0.076	0.332	4.761	4.194

JRT3T35	blood/lymph	0.079	0.124	7.610	96.813
EB3	blood/lymph	0.083	891.047	25.739	104.271
DEL	blood/lymph	0.089	2024.822	8.091	148.056
MOLM13	blood/lymph	0.089	0.659	5.041	1.727
BC1	blood/lymph	0.091	76.186	26.256	58.809
JEKO1	blood/lymph	0.098	2.718	1.402	1.966
RPMI8866	blood/lymph	0.100	3.015	0.266	0.635
CROAP2	blood/lymph	0.101	1.363	4.170	2.806
A3KAW	blood/lymph	0.102	188.901	16.825	240.363
CA46	blood/lymph	0.115	154.631	8.848	12.762
AMO1	blood/lymph	0.119	11.791	6.788	6.034
BL70	blood/lymph	0.121		3.630	7.916
RPMI8226	blood/lymph	0.121	20.862	9.733	120.000
MV411	blood/lymph	0.121	1.908	13.662	4.072
DB	blood/lymph	0.124		14.954	175.601
JIYOYEP2003	blood/lymph	0.126	214.340	17.036	24.798
A4FUK	blood/lymph	0.128	0.855	10.424	14.779
MCCAR	blood/lymph	0.135	0.146	179.217	13.090
NKM1	blood/lymph	0.135	11.410	3.911	2.997
U698M	blood/lymph	0.138	29.797	21.165	88.321
HC1	blood/lymph	0.146	92.931	3.848	3.709
REH	blood/lymph	0.149	1.836	0.850	0.666
KG1	blood/lymph	0.151	113.113	32.798	9.277
KASUMI1	blood/lymph	0.153	0.499	0.911	0.437
MOLP8	blood/lymph	0.160	2.535	3.359	3.786
MONOMAC6	blood/lymph	0.166	1.455	4.420	2.518
EOL1	blood/lymph	0.168	0.905	24.014	5.560
DG75	blood/lymph	0.172	4.847	54.735	155.821
KU812	blood/lymph	0.178	7.788	48.495	1086.897
SUDHL16	blood/lymph	0.178	0.505	3.235	1.285
DAUDI	blood/lymph	0.179	39.470	5.957	10.877
RAJI	blood/lymph	0.181	716.889	58.906	157.034
WSUNHL	blood/lymph	0.185	0.344	0.149	0.247
MOLT16	blood/lymph	0.186	0.025	43.151	8.730
L540	blood/lymph	0.188	61.509	43.411	10.889
LOUCY	blood/lymph	0.197	0.147	139.459	3.362
BE13	blood/lymph	0.198	11.073	305.799	141.762
GDM1	blood/lymph	0.218	1027.702	2.986	1.282
HAL01	blood/lymph	0.222	5.195	0.660	0.250
MOLT13	blood/lymph	0.228	34.297	58.799	276.863
OCILY7	blood/lymph	0.249	0.076	2.015	7.747
RCHACV	blood/lymph	0.255	0.910	0.655	0.715
KMOE2	blood/lymph	0.258	6.687	25.880	11.267
ARH77	blood/lymph	0.258	1821.187	7.327	10.417
SKMM2	blood/lymph	0.260	23.948	23.980	5.197
OCIAML2	blood/lymph	0.264	42.213	31.289	27.789
MEG01	blood/lymph	0.267	141.030	123.585	119.371

KMS11	blood/lymph	0.267	295.708	7.989	4.970
MC1010	blood/lymph	0.270	0.686	0.362	0.629
JURKAT	blood/lymph	0.271	7.445	26.197	11.429
KARPAS620	blood/lymph	0.274	0.473	10.986	6.115
H9	blood/lymph	0.285	42.140	74.904	36.009
JVM2	blood/lymph	0.292	5.080	0.834	2.045
HDMYZ	blood/lymph	0.298	118.538	17.411	35.880
SR	blood/lymph	0.304	2.009	20.767	9.299
SCI1	blood/lymph	0.323	3559.114	23.254	129.733
ATN1	blood/lymph	0.342	2.783	6.219	2.427
JJN3	blood/lymph	0.358	9.531	189.519	78.551
NOMO1	blood/lymph	0.367	4.286	13.741	4.725
MN60	blood/lymph	0.379	242.568	37.411	28.227
BV173	blood/lymph	0.385	2.686	171.246	16.904
ML2	blood/lymph	0.386	12.464	1.999	5.795
OCIAML3	blood/lymph	0.394	7.274	8.069	2.138
L1236	blood/lymph	0.395	3.133	16.137	17.078
JVM3	blood/lymph	0.405	6.837	22.366	19.983
KE37	blood/lymph	0.424	0.173	164.312	337.216
VAL	blood/lymph	0.448	241.688	14.824	33.459
WIL2NS	blood/lymph	0.461	36.669	31.636	23.199
KOPN8	blood/lymph	0.473		41.143	266.524
ALLSIL	blood/lymph	0.484		6.723	4.755
QIMRWIL	blood/lymph	0.491	31.746	5.716	25.875
K562	blood/lymph	0.499	53.881	135.178	773.969
NB4	blood/lymph	0.510	18.251	4.907	2.490
CESS	blood/lymph	0.525	59.284	3.623	8.225
BL41	blood/lymph	0.544	3.454	22.007	70.892
P32ISH	blood/lymph	0.544	8.026	6.395	9.086
CMLT1	blood/lymph	0.584	130.211	152.273	942.283
PL21	blood/lymph	0.653	1.083	7.241	2.389
KARPAS45	blood/lymph	0.685	2.175	1.906	23.922
NALM6	blood/lymph	0.691	2.052	3.270	3.536
JSC1	blood/lymph	0.695	13.649	246.620	189.798
SKM1	blood/lymph	0.722	1.453	1.928	1.776
EJM	blood/lymph	0.753	33.121	23.746	111.305
NAMALWA	blood/lymph	0.757	63.992	15.219	7.873
TK	blood/lymph	0.757	29.973	20.978	63.742
KARPAS231	blood/lymph	0.763	1.616	3.168	11.618
CMK	blood/lymph	0.768	105.408	68.138	42.115
CTV1	blood/lymph	0.771	2.899	18.493	22.438
KMH2	blood/lymph	0.802	23.928	41.132	64.305
KARPAS299	blood/lymph	0.820	57.146	83.847	165.436
JURLMK1	blood/lymph	0.827	6.538	90.881	202.677
LC41	blood/lymph	0.835	191.636	52.465	171.039
MC116	blood/lymph	0.851	892.108	20.367	86.809
HT	blood/lymph	0.852	1.174	6.688	23.004

SUPHD1	blood/lymph	0.885	5.543	31.992	73.944
SUDHL4	blood/lymph	0.893	0.238	1.012	458.613
DND41	blood/lymph	0.912	40.063	24.543	87.633
SUDHL8	blood/lymph	0.918	70.804	64.530	270.072
OCILY19	blood/lymph	0.962	18.448	13.133	37.825
HEL	blood/lymph	1.023	1370.458	306.918	600.188
P31FUJ	blood/lymph	1.100	3.207	184.763	16.751
CTB1	blood/lymph	1.134	11.693	16.675	307.773
RS411	blood/lymph	1.173	2.501	34.067	98.665
IM9	blood/lymph	1.184	1064.479	348.479	159.194
KARPAS1106P	blood/lymph	1.193	5.811	15.941	315.771
OCIM1	blood/lymph	1.259	450.151	222.589	847.650
SET2	blood/lymph	1.359	36.027	158.881	35.886
ST486	blood/lymph	1.365	112.482	225.841	1748.013
LP1	blood/lymph	1.432	16.174	114.760	289.315
OPM2	blood/lymph	1.621	2.499	139.191	387.894
OCIAML5	blood/lymph	1.705	386.588	5.672	2.437
RL	blood/lymph	2.182	12.663	4.445	37.120
RPMI6666	blood/lymph	2.216	4.096	10.551	14.758
L428	blood/lymph	2.298	1592.791	133.557	408.307
JM1	blood/lymph	2.418	79.891	35.655	46.144
SUPB8	blood/lymph	2.596	15.504	91.747	960.551
GRANTA519	blood/lymph	2.801	30.580	114.589	54.760
SUDHL10	blood/lymph	2.805	0.395	12.216	23.354
U266	blood/lymph	3.330		40.322	348.119
P12ICHIKAWA	blood/lymph	3.347	18.252	149.770	83.434
MLMA	blood/lymph	3.730	58.228	7.291	3.691
EB2	blood/lymph	3.885	53.938	31.059	70.204
PF382	blood/lymph	4.301	4.856	170.133	111.204
YT	blood/lymph	4.548	53.347	136.455	709.283
SLVL	blood/lymph	4.966	59.456	36.874	61.930
LAMA84	blood/lymph	4.968	190.251	60.663	580.681
KARPAS422	blood/lymph	5.090	2.809	1.839	25.690
BALL1	blood/lymph	5.440	74.328	51.484	634.828
HS445	blood/lymph	6.722		5.603	1.818
NUDUL1	blood/lymph	7.095	0.355	17.071	37.439
HDLM2	blood/lymph	7.185	34.375	73.058	165.795
MOT	blood/lymph	7.226	7.381	32.218	47.028
EHEB	blood/lymph	7.494	2.206	0.350	27.656
ME1	blood/lymph	7.599	2814.551	191.303	1974.787
SUPT1	blood/lymph	8.984	5.558	364.567	294.388
ALLPO	blood/lymph	9.273	1.611	50.405	147.828
THP1	blood/lymph	10.825	22.681	16.738	25.681
HL60	blood/lymph	23.531	8.015	31.594	47.828
MHHCALL2	blood/lymph	27.819	169.518	133.014	739.901
ROS50	blood/lymph	43.499	590.362	118.654	443.063
KY821	blood/lymph	43.582	13.022	12.869	18.201

RCK8	blood/lymph	261.043	1901.977	139.477	474.596
WSUDLCL2	blood/lymph	536.807	0.003	37.875	781.327
FARAGE	blood/lymph	1044.822	0.839	286.282	1128.638
MG63	bone	0.088	3.974	0.840	8.225
CHSA0108	bone	0.112	9.827	5.345	7.539
ES7	bone	0.123	16.606	0.445	6.472
EW7	bone	0.179	5.481	5.263	0.717
ES1	bone	0.221	11.119	30.662	3.972
CAL72	bone	0.279	99.802	85.533	65.185
SKES1	bone	0.285	5.283	28.108	8.026
EW3	bone	0.293	12.910	5.933	2.196
ES5	bone	0.299	8.125	6.572	2.859
TC71	bone	0.322	58.647	11.373	2.391
ES8	bone	0.328	13.028	15.631	7.103
ES3	bone	0.365		11.516	5.899
SKPNDW	bone	0.378		21.681	52.993
MHHES1	bone	0.380	46.221	17.105	12.150
CHSA0011	bone	0.491		5.802	24.713
EW13	bone	0.536	5.342	98.001	66.402
EW18	bone	0.543	0.334	10.650	7.302
EW1	bone	0.546	465.618	37.839	13.638
G292CLONEA141B1	bone	0.562		3.741	2.750
U2OS	bone	0.579	7.759	11.282	10.524
ES4	bone	0.723	19.587	8.805	1.977
EW22	bone	0.732	28.268	12.877	17.031
HUO9	bone	0.786	6.229	2.681	1.786
EW24	bone	0.945	121.826	86.182	7.522
HOS	bone	0.985	2.734	37.474	13.257
CHSA8926	bone	1.110	235.426	3.746	22.274
ES6	bone	1.113	55.696	120.021	40.917
SARC9371	bone	1.245	14.636	14.397	40.393
CS1	bone	1.607	9.220	245.358	70.119
CADOES1	bone	1.656	837.943	166.503	348.356
SJSA1	bone	1.804	30.770	22.079	35.156
NOS1	bone	1.907	103.782	26.512	5.402
NY	bone	3.787	46.740	315.298	41.071
SAOS2	bone	5.105	36.803	10.616	17.085
CAL78	bone	6.944	384.381	69.640	139.028
HUO3N1	bone	11.092	21.160	9.123	19.038
EW11	bone	521.615	6.617	29.999	1280.143
SF295	brain	0.175	3.897	9.591	42.371
D283MED	brain	0.176	52.141	46.169	41.886
T98G	brain	0.200	17.652	2.341	5.329
D263MG	brain	0.203	63.524	4.385	12.206
GI1	brain	0.207	2.145	6.222	10.270
DAOY	brain	0.219	24.768	51.606	23.587
H4	brain	0.232	1.042	2.293	9.882

GMS10	brain	0.268	19.764	3.119	8.613
GAMG	brain	0.334	1.971	1.213	8.163
SKMG1	brain	0.431	7.137	3.317	4.608
SW1088	brain	0.445	2.207	1.166	48.659
MOGGUVW	brain	0.450	5.948	6.409	27.190
SF126	brain	0.457	12.893	22.098	9.535
KNS42	brain	0.474	96.734	131.150	75.469
KNS81FD	brain	0.479	11.435	11.521	72.281
MOGGCCM	brain	0.481	10.979	3.404	55.769
BECKER	brain	0.487	5.976	3.383	10.842
ONS76	brain	0.505	26.471	89.477	105.283
CCFSTTG1	brain	0.520	34.146	13.821	21.951
D566MG	brain	0.557	2.920	4.570	27.457
PFSK1	brain	0.561		28.275	15.552
8MGBA	brain	0.578	6.760	6.012	9.249
LN405	brain	0.644		3.692	74.054
42MGBA	brain	0.660	1.564	4.103	5.778
D423MG	brain	0.719	19.949	4.688	13.730
SNB75	brain	0.748	32.766	2.585	4.138
YKG1	brain	0.819	5.338	17.284	23.132
SF539	brain	0.888	3.296	29.369	28.829
U251	brain	0.936	13.719	189.285	81.603
LN18	brain	1.000	97.010	0.805	196.851
YH13	brain	1.042	2.222	1.847	10.861
LNZTA3WT4	brain	1.046	51.291	72.752	20.929
KALS1	brain	1.169	13.233	1.726	86.755
KS1	brain	1.181	21.500	4.436	10.921
A172	brain	1.188	83.319	22.948	310.315
SW1783	brain	1.193	43.450	9.266	161.772
DBTRG05MG	brain	1.311	74.007	10.715	13.188
LN229	brain	1.347	53.206	38.769	31.438
GB1	brain	1.405	14.801	28.637	15.997
CAS1	brain	1.541	15.143	12.546	202.522
SF268	brain	1.728	61.781	15.787	61.087
M059J	brain	1.736	8.208	13.795	109.112
D392MG	brain	1.910	28.369	40.602	56.878
NMCG1	brain	2.045	191.437	43.983	12.684
D542MG	brain	2.084	47.953	10.546	64.499
D502MG	brain	2.287	133.156	3.267	12.577
NO10	brain	2.700	54.143	10.385	228.901
DKMG	brain	2.770	9.356	23.560	162.357
D247MG	brain	2.927	121.580	12.748	43.250
KINGS1	brain	3.043	16.687	50.345	31.262
D336MG	brain	4.039	3.777	2.351	8.594
HS683	brain	4.457	33.037	9.870	147.811
NO11	brain	4.725	240.715	5.222	67.832
AM38	brain	7.091	65.013	247.767	1251.152

U118MG	brain	12.308	10.091	41.053	17.532
U87MG	brain	14.772	21.068	15.726	29.325
HCC1500	breast	0.109	355.730	144.857	637.185
MRKNU1	breast	0.114	368.298	158.508	897.907
OCUBM	breast	0.187	0.094	18.953	1.079
CAL120	breast	0.223	8.381	10.650	10.226
EVSAT	breast	0.225	0.147	0.018	7.855
MFM223	breast	0.255	0.949	59.808	4.409
CAL51	breast	0.280	0.762	46.293	255.380
BT549	breast	0.379	7.902	1.054	59.864
JIMT1	breast	0.397	4.020	7.305	2.316
MDAMB330	breast	0.408		14.624	0.888
MCF7	breast	0.500	3.189	3.629	4.459
HCC1143	breast	0.576	156.047	75.746	13.869
MB157	breast	0.658		12.850	13.775
DU4475	breast	0.730	8.312	80.189	36.344
HCC1395	breast	0.954	66.271	2.749	45.814
HS578T	breast	0.978	5.228	6.452	53.923
EFM192A	breast	1.002	0.939	14.228	5.288
HCC2157	breast	1.111	0.071	2.689	30.581
HCC1806	breast	1.129	103.984	291.343	52.769
MDAMB361	breast	1.240	0.725	6.480	1.066
MDAMB453	breast	1.263		12.820	0.830
MDAMB468	breast	1.328	31.029	9.925	320.560
MDAMB231	breast	1.359	67.541	8.663	28.544
UACC893	breast	1.583	0.152	92.878	4.416
EFM19	breast	1.615	2.849	3.561	2.857
HCC1599	breast	1.735		0.483	5.488
HCC70	breast	1.754	2.637	0.251	47.609
MDAMB436	breast	1.859	106.943	1.530	108.859
MDAMB415	breast	1.964		0.428	30.702
HCC1954	breast	1.991	0.743	71.447	3.856
BT20	breast	2.217	5.614	33.123	2.907
CAL851	breast	2.294	1383.848	8.880	48.352
CAMA1	breast	2.392	1.165	9.955	897.853
AU565	breast	2.437	2.893	13.665	12.465
HCC2218	breast	3.052	47.220	14.464	44.690
MDAMB175VII	breast	3.066	0.670	128.237	9.968
HCC1187	breast	3.156	11.683	11.297	22.816
HCC202	breast	3.789	207.347	12.899	2.975
T47D	breast	3.962	11.446	7.513	11.670
HCC1419	breast	4.064	1.868	90.223	21.284
HCC1428	breast	4.089	4884.748	158.278	171.722
COLO824	breast	4.186		8.174	10.366
HCC1937	breast	4.189	95.438	16.226	243.376
HCC38	breast	4.193	113.651	37.035	96.548
BT483	breast	4.283	899.749	47.819	70.888

HDQP1	breast	5.089	72.459	9.370	117.643
BT474	breast	5.228	8.653	10.565	5.049
MDAMB157	breast	5.287	330.773	136.583	259.222
HCC1569	breast	5.664	2.599	17.641	24.495
ZR7530	breast	28.829	133.451	46.311	55.154
ME180	cervix	0.171	0.076	20.071	1.898
SISO	cervix	0.254	134.761	16.642	9.986
TCYIK	cervix	0.392	144.961	22.311	39.887
CASKI	cervix	0.399	10.530	5.406	4.151
C33A	cervix	0.508	10.462	46.516	21.513
OMC1	cervix	0.855	7.152	119.919	2.828
SKGIIIA	cervix	1.234	80.096	10.730	35.581
HELA	cervix	1.364	376.344	18.735	30.521
MS751	cervix	1.419	1447.653	6.581	38.156
SIHA	cervix	1.770		34.139	212.563
HT3	cervix	1.967	715.676	14.037	13.978
BOKU	cervix	2.444	127.720	17.210	22.261
C4I	cervix	6.997	98.965	8.787	80.485
SW756	cervix	13.053	819.465	11.166	55.796
DOTC24510	cervix	14.058	85.127	78.461	9.790
CL40	colon	0.118	6.819	4.054	1.034
MDST8	colon	0.132	13.133	10.601	9.118
SNUC1	colon	0.275	1.190	10.992	8.587
COLO741	colon	0.400	7.154	28.931	66.592
H716	colon	0.430	2.933	1.786	7.184
LOVO	colon	0.503		0.065	6.400
SW48	colon	0.518	8.455	5.723	1.817
COLO320HSR	colon	0.532	62.329	36.945	7.594
HCT116	colon	0.545	10.697	5.695	7.968
HCT15	colon	0.560	44.504	131.186	10.210
SW620	colon	0.657	237.074	30.449	64.159
RKO	colon	0.661	6.829	37.199	5.323
COLO205	colon	0.752	13.054	22.974	12.571
SNU81	colon	0.826	20.085	1.415	12.534
LS180	colon	0.966	163.176	115.085	6.886
HT115	colon	1.021	487.294	115.377	17.788
SKCO1	colon	1.041	80.431	16.863	3.126
OUMS23	colon	1.078	25.088	16.952	142.352
H508	colon	1.136	0.466	34.812	16.893
LS513	colon	1.369	5.766	67.342	16.122
CL11	colon	1.452	160.919	17.182	65.741
H747	colon	1.464	22.827	7.324	8.783
LS1034	colon	1.781	107.618	66.233	47.594
SNUC2B	colon	1.795	86.028	15.350	38.460
H630	colon	1.798	3038.885	67.000	23.883
SNU407	colon	1.847	14.270	86.038	4.308
LIM1215	colon	2.155	179.101	53.758	40.344

SNUC5	colon	2.331	2856.373	22.873	6.824
HUTU80	colon	2.400	31.032	98.716	168.551
HCC2998	colon	2.570	5.177	77.236	134.160
CL34	colon	3.008	1810.434	33.390	10.141
LS411N	colon	3.056	16.504	180.144	173.774
SW403	colon	3.199	22.296	47.500	17.774
DIFI	colon	3.625	5.750	86.555	7.068
CAR1	colon	3.968	28.978	17.245	45.082
SW837	colon	4.043	1986.328	25.572	41.623
C2BBE1	colon	5.200	60.224	30.545	148.013
SW1463	colon	6.040	4529.840	85.522	24.885
LS123	colon	6.912	87.966	5.969	46.005
GP5D	colon	7.027	12.303	77.543	4.199
HCC56	colon	9.894	260.639	101.664	28.478
HT55	colon	9.998	223.479	57.261	24.445
T84	colon	15.906	325.409	55.799	9.895
SW948	colon	17.024	426.963	72.949	11.436
SW1116	colon	18.928	165.317	61.790	99.742
CW2	colon	19.934	451.399	156.895	37.513
COLO678	colon	24.818	207.197	110.572	89.376
HT29	colon	29.396	157.593	23.929	14.426
KM12	colon	30.142	8.848	159.505	834.476
SNU1040	colon	32.145		264.825	378.921
CCK81	colon	59.719	79.800	198.314	14.703
RCM1	colon	106.735	33.822	92.834	33.955
SNU61	colon	111.320	238.730	178.515	168.259
SW1417	colon	113.035	87.742	36.179	143.245
U031	kidney	0.058	7.496	0.342	4.504
HA7RCC	kidney	0.091	116.964	1.498	6.838
G401	kidney	0.110	50.791	3.135	2.314
LB1047RCC	kidney	0.156	9.307	6.787	2.327
RCCMF	kidney	0.195	22.754	3.544	7.665
LB2241RCC	kidney	0.201	16.107	0.791	3.296
LB996RCC	kidney	0.214	0.887	0.874	4.656
SKNEP1	kidney	0.225	8.953	5.009	4.420
BB65RCC	kidney	0.268	155.082	5.713	10.666
RCCFG2	kidney	0.269	2.037	0.051	20.361
A498	kidney	0.271	5.736	0.124	370.017
SW13	kidney	0.288	23.204	21.496	56.125
RCCJF	kidney	0.308	2.590	0.252	9.882
RCCJW	kidney	0.326	13.619	1.218	7.302
ACHN	kidney	0.330	27.590	1.824	11.369
TK10	kidney	0.386	23.804	4.051	10.953
769P	kidney	0.448		1.835	39.014
BFTC909	kidney	0.463	3.826	10.184	6.749
RCCAB	kidney	0.465	134.204	30.956	31.059
OSRC2	kidney	0.506	55.635	0.695	134.974

KMRC1	kidney	0.545	3.050	1.215	516.200
SN12C	kidney	0.545	34.542	8.811	24.350
CAL54	kidney	0.588	48.939	0.484	7.182
CAKI1	kidney	0.677	9.784	1.143	46.547
RCCER	kidney	0.680	215.072	10.221	9.578
VMRCRCZ	kidney	0.701	16.635	5.595	12.666
7860	kidney	0.763	3402.178	7.324	353.770
NCC010	kidney	1.274	633.466	4.843	16.949
KMRC20	kidney	1.476	55.379	567.976	42.989
RXF393	kidney	1.521	5.868	1.001	43.659
SW156	kidney	1.769	3.781	16.670	40.753
RCC10RGB	kidney	2.088	407.085	8.437	19.510
NCC021	kidney	3.284	14.875	0.171	17.062
A704	kidney	4.306		11.110	17.321
HUH7	liver	0.062	21.313	15.037	8.655
HEP3B217	liver	0.150	2.359	25.930	9.373
JHH1	liver	0.152	1159.130	2.374	3.811
HLE	liver	0.155	107.089	16.205	58.107
JHH6	liver	0.208	12.051	10.221	15.922
HUH1	liver	0.272	16.712	107.873	16.980
SKHEP1	liver	0.298	113.445	8.740	31.288
JHH2	liver	0.325	7.424	0.211	8.852
JHH4	liver	0.331	1128.539	6.027	13.332
SNU423	liver	0.446	21.661	5.593	29.616
JHH7	liver	0.451	25.027	40.211	25.590
SNU398	liver	0.486		289.834	194.169
SNU182	liver	0.556	8.317	5.156	13.161
SNU387	liver	0.679	213.298	6.300	6.132
SNU449	liver	0.806	1172.743	13.612	11.304
HUH6CLONE5	liver	0.870	50.175	139.325	12.285
C3A	liver	4.293	69.418	96.879	297.299
H292	lung	0.013	3.222	1.305	1.960
H1417	lung	0.048	0.994	1.350	0.392
LU99A	lung	0.052	15.200	13.771	5.084
LXF289	lung	0.078	3.616	1.153	17.143
SKMES1	lung	0.084	432.708	0.611	3.083
H1793	lung	0.111	35.289	15.336	83.543
H1355	lung	0.119	111.465	8.551	16.096
HCC44	lung	0.120	1.080	1.288	9.761
H2030	lung	0.127	18.632	3.452	11.541
EBC1	lung	0.134	5.193	0.326	96.107
CORL32	lung	0.138		9.790	4.386
A549	lung	0.150	28.624	2.428	8.309
SBC1	lung	0.160	6.105	2.350	4.416
H2122	lung	0.166	400.830	57.315	12.984
H1993	lung	0.171	33.242	44.230	63.978
H1944	lung	0.175	59.496	2.275	2.028

H446	lung	0.176	1.746	0.239	12.752
MSTO211H	lung	0.180	950.608	9.138	20.570
H2452	lung	0.184	26.572	4.757	35.990
H647	lung	0.208	6.286	9.910	5.076
H1975	lung	0.220	2.798	5.170	3.388
H1299	lung	0.228	75.436	1.061	26.008
H1648	lung	0.228	14.638	34.061	6.338
H838	lung	0.239		7.805	10.076
H2023	lung	0.257	48.894	2.235	8.271
H1666	lung	0.258	61.316	2.102	4.520
HCC15	lung	0.259	28.812	19.363	32.745
H2373	lung	0.269	2.710	7.657	12.376
H290	lung	0.280	61.965	32.179	67.688
H2347	lung	0.292	107.647	19.818	61.638
H524	lung	0.325	72.699	36.039	35.773
SBC3	lung	0.326	212.661	34.152	43.134
KNS62	lung	0.330	145.768	24.647	569.254
H2405	lung	0.340	110.180	8.299	59.701
H1341	lung	0.345	10.594	2.588	1.116
DMS114	lung	0.353	23.325	24.102	6.386
H1876	lung	0.355	30.165	15.696	38.780
H2461	lung	0.359	285.055	10.370	8.018
LK2	lung	0.361	135.396	11.147	16.225
H1568	lung	0.366	44.965	15.824	8.903
H1581	lung	0.387	3329.126	91.833	823.480
CAL12T	lung	0.399	119.600	10.326	16.190
H2803	lung	0.399	84.014	43.244	109.149
H2342	lung	0.410	284.243	6.668	20.285
H28	lung	0.442	21.180	38.014	10.207
H2591	lung	0.451	70.036	22.088	27.132
H2228	lung	0.455		9.208	11.480
H460	lung	0.455	1.739	70.146	15.508
H2369	lung	0.495	896.960	84.414	153.715
LU139	lung	0.504	534.114	573.760	830.617
H2126	lung	0.507	53.238	6.705	13.486
PC14	lung	0.511	17.630	17.295	6.887
SW1573	lung	0.522	0.827	7.017	4.792
H661	lung	0.546	18.675	6.206	17.650
H2170	lung	0.569	21.032	39.916	13.106
H209	lung	0.601	294.933	199.551	114.974
H2722	lung	0.609	40.235	36.160	127.895
H69	lung	0.613	12.140	4.979	1.560
SBC5	lung	0.639	8.641	40.487	17.640
EPLC272H	lung	0.645	4.702	4.385	5.222
SKLU1	lung	0.647	64.542	57.705	74.556
RERFLCMS	lung	0.649	6.854	3.096	4.404
H1048	lung	0.654	14.881	6.446	1.368

H82	lung	0.676	13.313	82.806	49.939
H1755	lung	0.709	5.482	10.317	9.467
MPP89	lung	0.730	40.553	10.440	140.560
H847	lung	0.780	105.734	23.514	7.791
H1792	lung	0.791	374.473	6.929	6.878
H2818	lung	0.830	79.670	113.268	529.212
H2085	lung	0.842	63.100	0.178	16.114
H1437	lung	0.872	22.667	157.635	20.608
H1155	lung	0.878	1.467	34.442	359.158
H2110	lung	0.882	3.665	12.730	9.293
CORL105	lung	0.882	34.755	13.125	360.438
H1703	lung	0.903	12.944	3.916	3.823
H2009	lung	0.905	23.380	34.911	13.832
H2731	lung	0.914	57.284	92.960	20.839
HCC78	lung	0.916		1.065	3.022
HOP92	lung	0.945	12.978	3.181	10.493
HOP62	lung	0.958	44.963	91.603	70.952
CPCN	lung	0.976	71.026	6.793	18.863
CALU3	lung	1.083	94.518	38.888	7.835
H2795	lung	1.101	37.750	12.225	30.741
LOUNH91	lung	1.104	21.646	11.619	5.673
LCLC97TM1	lung	1.112	322.464	7.006	10.136
RERFLCSQ1	lung	1.131	5.670	22.196	3.520
H1781	lung	1.150	156.123	42.906	114.061
H211	lung	1.175	2050.992	46.033	227.445
UMC11	lung	1.221	720.399	44.805	613.683
H1650	lung	1.262	103.254	6.612	27.365
SW900	lung	1.305	227.897	19.175	14.102
IALM	lung	1.341	1589.562	64.215	45.266
H841	lung	1.360	50.832	16.218	26.080
LB647SCLC	lung	1.393	66.869	34.018	49.707
CORL23	lung	1.403	17.900	51.543	46.245
DMS273	lung	1.436	0.554	10.769	402.953
HARA	lung	1.495	3.140	41.183	17.923
CALU6	lung	1.525	410.833	101.239	42.773
H358	lung	1.538	251.124	18.453	11.219
H720	lung	1.569	0.285	25.709	3.844
ABC1	lung	1.592	113.485	11.316	32.110
H1623	lung	1.596	5.957	9.264	8.912
H2286	lung	1.648	120.722	14.770	90.136
H2869	lung	1.682	507.268	133.901	41.837
CHAGOK1	lung	1.697	51.595	10.162	72.483
H1770	lung	1.701	1.090	30.198	114.193
ISTMES1	lung	1.751	254.907	215.773	43.771
H1105	lung	1.827		50.323	257.696
H3255	lung	1.847	50.747	26.700	14.657
H1915	lung	1.873	708.645	176.214	743.090

H1869	lung	1.878	114.992	8.735	302.362
H146	lung	1.936	2436.042	75.191	388.010
ISTSL1	lung	1.953	45.183	69.450	542.472
H1395	lung	2.037	7.420	18.369	174.671
ISTSL2	lung	2.048	0.709	109.806	255.336
H596	lung	2.063	48.939	8.040	12.735
VMRCLCD	lung	2.135	831.414	168.962	910.679
H2595	lung	2.163	1223.866	20.412	68.506
H1436	lung	2.256		72.952	681.431
H23	lung	2.312	31.129	95.446	25.265
PC3_[JPC3]	lung	2.325	12.653	48.289	25.786
H3122	lung	2.329	40.339	156.017	77.912
A427	lung	2.369	5.583	72.831	78.171
BEN	lung	2.374	43.482	205.846	311.809
H513	lung	2.533	2752.922	17.635	55.160
H2810	lung	2.540	13.613	25.922	26.540
H650	lung	2.686	1923.377	203.761	21.587
CORL88	lung	2.691	338.172	46.624	345.927
HCC827	lung	2.817	99.357	197.560	9.320
H522	lung	2.820	102.862	141.841	38.683
MS1	lung	2.950	19.791	28.077	55.473
H520	lung	2.980	23.807	6.064	250.932
DMS53	lung	2.989	1396.449	335.385	934.649
H2227	lung	3.058	271.099	20.327	121.939
H2087	lung	3.123	5577.428	248.573	33.527
SW1271	lung	3.125	126.390	216.540	115.486
LC2AD	lung	3.134	25.394	12.436	7.462
SHP77	lung	3.389	2504.056	276.098	1186.503
H810	lung	3.428	192.136	255.356	216.588
H1651	lung	3.453	137.854	35.556	331.082
LC1SQ	lung	3.471	70.051	38.595	16.980
H727	lung	3.488	213.013	204.561	126.123
H820	lung	3.540		1.437	33.710
RERFLCKJ	lung	3.848	115.550	13.772	59.356
EKVX	lung	4.317	71.292	2.497	10.761
HCC366	lung	4.331	193.341	13.768	9.791
H1694	lung	4.344	87.208	207.942	515.580
H2804	lung	4.393	30.095	151.831	29.535
SCLC21H	lung	4.458	24.306	159.591	136.082
H2444	lung	5.042	1243.096	34.610	94.975
H226	lung	5.307	46.487	53.479	16.203
H1573	lung	5.527	150.712	54.274	122.788
LU134A	lung	5.895	2.759	19.990	50.738
CORL321	lung	6.186	166.936	41.387	204.219
H835	lung	6.392	16.647	28.028	314.956
LU165	lung	6.468	1805.954	235.679	268.050
H2172	lung	6.678	43.590	123.083	72.455

H526	lung	7.644	4.261	203.012	1429.607
H2291	lung	8.053	47.382	3.315	47.725
COLO668	lung	8.334	4009.922	72.292	160.822
H1836	lung	8.583	2913.254	48.478	173.584
H1563	lung	8.757	31.480	15.325	17.832
H196	lung	9.849	40.535	65.792	0.562
H1838	lung	11.262	93.316	45.916	57.884
LU135	lung	11.307	22.307	50.214	358.111
H441	lung	13.888	399.513	19.986	82.222
H1435	lung	17.112	257.573	32.437	880.310
H2141	lung	17.888	464.325	191.785	208.226
H2066	lung	19.206	23.460	2.648	29.202
LCLC103H	lung	20.266	13.524	250.653	434.091
H1688	lung	22.262	19.152	24.722	122.591
H1092	lung	27.766	177.128	166.704	299.255
CORL95	lung	28.613	120.427	48.150	210.401
H187	lung	31.080	14.182	40.743	175.879
H322M	lung	39.285	624.322	44.689	131.987
H2029	lung	49.186	389.311	140.641	375.087
H345	lung	57.048	431.806	43.532	217.119
DMS79	lung	60.778	97.403	72.371	416.866
LU65	lung	95.426	3633.243	52.513	125.434
H2081	lung	111.971	319.279	173.821	1013.922
H1304	lung	120.919	35.794	57.857	113.505
H2135	lung	1728.529	3666.834	304.223	420.147
STS0421	muscle (not otherwise specified)	0.232	11.185	6.576	12.576
G402	muscle (smooth)	0.083	6.633	5.587	27.287
SKLMS1	muscle (smooth)	0.212	10.774	1.130	6.246
RKN	muscle (smooth)	0.606	14.927	4.704	16.527
A204	muscle (striated)	0.058	4.039	0.740	2.294
RH41	muscle (striated)	0.165	0.631	26.651	3.419
KYM1	muscle (striated)	0.260	1.729	10.460	16.805
A673	muscle (striated)	0.689	27.712	44.849	7.006
SJRH30	muscle (striated)	0.885	1.260	18.697	2.618
RD	muscle (striated)	1.123	239.412	17.892	4.662
RH18	muscle (striated)	8.562	31.720	11.090	23.565
NB69	nervous system (PNS)	0.045	55.410	2.992	5.413
KELLY	nervous system (PNS)	0.117	124.103	120.267	59.961
NBSUSSR	nervous system (PNS)	0.174	3.974	3.982	2.463
NB12	nervous system (PNS)	0.263	12.458	1.656	41.283
NB5	nervous system (PNS)	0.281	40.541	6.667	7.053
GIMEN	nervous system (PNS)	0.340	7.340	5.937	12.666
CHP212	nervous system (PNS)	0.361	15.803	8.483	2.058
NB7	nervous system (PNS)	0.455	15.062	5.059	12.847
BE2M17	nervous system (PNS)	0.580	85.831	39.909	8.169
SKNAS	nervous system (PNS)	0.589	38.015	172.263	58.221
ACN	nervous system (PNS)	0.618	5.577	70.003	20.147

GOTO	nervous system (PNS)	0.861	47.293	63.007	11.303
TASK1	nervous system (PNS)	0.936	916.658	17.838	15.662
NB10	nervous system (PNS)	1.136	114.170	27.812	7.615
NB1	nervous system (PNS)	1.309	2.554	1.240	3.175
CHP126	nervous system (PNS)	1.643	119.273	72.412	92.927
NH12	nervous system (PNS)	1.732	95.432	15.789	10.889
IMR5	nervous system (PNS)	1.794	363.153	68.915	112.372
MCIXC	nervous system (PNS)	1.888	21.435	123.369	27.211
NB14	nervous system (PNS)	2.444	17.143	61.908	109.885
SKNFI	nervous system (PNS)	2.847		79.656	16.110
SKNSH	nervous system (PNS)	3.071	19.433	40.275	5.938
NB13	nervous system (PNS)	5.032	74.402	159.491	203.424
TGW	nervous system (PNS)	7.622	7.056	162.502	63.074
SKNDZ	nervous system (PNS)	10.563	48.837	91.690	290.391
NB17	nervous system (PNS)	12.347	25.676	31.231	17.540
SIMA	nervous system (PNS)	22.881	74.902	95.863	29.258
LAN6	nervous system (PNS)	33.276	43.796	88.186	46.054
NBTU110	nervous system (PNS)	87.707	1850.568	395.865	1324.598
NB6	nervous system (PNS)	113.303	238.561	203.392	24.402
KPNYN	nervous system (PNS)	252.797		81.512	109.556
KYAE1	oesophagus	0.119	0.134	2.103	1.272
BB49HNC	oesophagus	0.124	5.274	0.011	1.187
OE21	oesophagus	0.135	3.898	17.988	3.870
JHU029	oesophagus	0.139	49.285	5.596	1.332
SKGT4	oesophagus	0.159	3.612	6.983	1.292
LB771HNC	oesophagus	0.184	2.567	11.375	6.288
HCE4	oesophagus	0.199	99.485	1.523	8.824
ECGI10	oesophagus	0.214	6.225	21.527	4.823
PCI4B	oesophagus	0.231	6.298	26.907	25.484
TE5	oesophagus	0.232	50.988	18.280	4.459
EMCBAC2	oesophagus	0.250	25.098	10.125	10.159
JHU028	oesophagus	0.281	89.706	16.931	10.555
JHU022	oesophagus	0.323	38.004	30.448	0.801
KYSE180	oesophagus	0.325	13.442	30.608	12.309
JHU011	oesophagus	0.327	31.859	141.442	9.234
KYSE410	oesophagus	0.407	61.349	141.740	7.173
KYSE510	oesophagus	0.415	39.617	5.726	6.319
TE4	oesophagus	0.458	2.080	31.265	16.371
FLO1	oesophagus	0.461	19.498	3.198	12.487
KYSE450	oesophagus	0.500	26.398	9.605	4.985
BB30HNC	oesophagus	0.628	39.227	50.390	6.940
DETROIT562	oesophagus	0.645	4.269	45.544	4.779
TE9	oesophagus	0.711	21.073	32.740	8.802
TT	oesophagus	0.749	38.156	68.232	6.901
TT	oesophagus	0.749	38.156	68.232	6.901
OACM51	oesophagus	0.790	26.141	17.994	90.795
KYSE270	oesophagus	0.802	107.015	7.428	6.895

TE10	oesophagus	1.012	9.945	22.588	10.380
ESO26	oesophagus	1.039	5.147	11.857	39.920
EMCBAC1	oesophagus	1.080	106.186	29.983	47.830
KYSE150	oesophagus	1.176	20.875	353.543	28.268
TE11	oesophagus	1.256	28.772	62.686	5.912
KYSE30	oesophagus	1.263	28.151	10.054	24.036
OE33	oesophagus	1.331	854.433	38.447	60.446
OACP4C	oesophagus	1.449	3.996	16.191	2.895
ESO51	oesophagus	1.514		148.636	20.293
KYSE140	oesophagus	1.561	1.369	144.534	113.321
TE12	oesophagus	1.630	41.272	4.848	5.223
TE8	oesophagus	1.749	6.011	18.582	5.813
KYSE520	oesophagus	1.808	76.035	170.266	15.169
A253	oesophagus	2.111	3.425	151.048	584.046
KYSE220	oesophagus	2.478	4.435	27.891	4.048
FADU	oesophagus	2.559	17.730	20.550	19.370
TE1	oesophagus	3.032	172.221	26.770	13.151
KYSE70	oesophagus	3.189	21.687	56.894	14.363
TE15	oesophagus	4.085	76.227	98.483	18.439
TE6	oesophagus	4.891	102.469	41.421	33.856
OE19	oesophagus	6.701	420.372	30.391	24.358
COLO680N	oesophagus	7.100	103.191	237.057	593.985
SCC90	oesophagus	7.756	4.792	55.503	35.479
UDSCC2	oesophagus	21.176	146.661	109.881	425.349
KYSE50	oesophagus	184.049	95.888	111.348	250.102
KON	oral/nasal cavity	0.040	10.951	38.785	7.930
HSC3	oral/nasal cavity	0.088	9.419	8.437	6.377
BICR22	oral/nasal cavity	0.120	8.148	6.167	6.630
DOK	oral/nasal cavity	0.201	1.531	2.749	1.185
SAT	oral/nasal cavity	0.218	8.303	9.732	3.312
HSC4	oral/nasal cavity	0.247	3.122	17.616	3.520
PECAPJ15	oral/nasal cavity	0.255	55.918	14.673	5.940
OSC20	oral/nasal cavity	0.261	2.474	10.633	10.734
CA922	oral/nasal cavity	0.317	8.312	23.620	6.629
HO1N1	oral/nasal cavity	0.338	5.954	9.431	6.799
HO1U1	oral/nasal cavity	0.371	1.946	34.260	10.012
CAL33	oral/nasal cavity	0.381	7.793	5.536	0.707
BICR10	oral/nasal cavity	0.400	6.214	3.965	2.511
BHY	oral/nasal cavity	0.533	181.677	11.008	15.221
OSC19	oral/nasal cavity	0.534	112.292	21.130	10.054
JHU019	oral/nasal cavity	0.626	8.399	1.323	97.677
HN	oral/nasal cavity	0.654	2.028	27.331	7.896
SCC15	oral/nasal cavity	0.663	230.263	3.328	4.350
CAL27	oral/nasal cavity	0.732	21.484	5.000	3.434
BICR78	oral/nasal cavity	0.745	38.732	5.969	4.805
SAS	oral/nasal cavity	0.786	43.511	90.462	30.164
KOSC2	oral/nasal cavity	0.810	14.205	76.869	10.808

PCI38	oral/nasal cavity	0.833	4.204	154.869	14.761
RPMI2650	oral/nasal cavity	0.852	115.811	36.690	23.555
SCC9	oral/nasal cavity	0.862	72.546	1.524	4.143
H3118	oral/nasal cavity	1.189	46.545	6.133	3.451
HSC2	oral/nasal cavity	1.284		25.571	2.276
SKN3	oral/nasal cavity	1.841	75.984	68.859	13.829
SCC25	oral/nasal cavity	1.952	12.330	20.857	7.359
PCI30	oral/nasal cavity	2.319	58.548	26.735	35.644
PCI15A	oral/nasal cavity	2.713	4.005	166.850	17.149
SCC4	oral/nasal cavity	4.995	25.251	80.036	16.267
PCI6A	oral/nasal cavity	27.231	10.156	74.280	17.151
KGN	ovary	0.109	176.853	3.915	23.918
OVCA420	ovary	0.119	99.890	2.233	1.925
HEY	ovary	0.129	16.500	158.102	8.615
OAW42	ovary	0.152	36.879	1.898	0.837
A2780	ovary	0.160	1.632	4.573	2.733
TOV112D	ovary	0.163	14.609	9.022	30.828
IOSE7516SV40	ovary	0.217	9.031	43.841	213.832
IGROV1	ovary	0.224	0.958	1.311	0.676
TYKNU	ovary	0.251	9.537	129.881	75.167
TOV21G	ovary	0.262	0.721	34.010	6.885
OVCAR5	ovary	0.264	28.698	5.003	6.852
OVCA433	ovary	0.271	43.286	5.542	5.739
OVCAR4	ovary	0.349	656.836	24.009	59.160
OC314	ovary	0.363	13.061	6.100	10.211
OVK18	ovary	0.401	0.696	8.436	28.502
OV7	ovary	0.409	9.274	0.820	15.219
OVTOKO	ovary	0.426	5.029	1.990	7.412
IOSE364	ovary	0.434	4.823	46.061	49.446
SKOV3	ovary	0.447	8.315	15.878	2.363
OVCAR3	ovary	0.490	170.377	4.850	36.488
DOV13	ovary	0.503	39.541	19.231	12.979
JHOS2	ovary	0.569	65.460	2.788	3.221
JHOS4	ovary	0.609	822.076	4.344	4.531
OVMIU	ovary	0.623	23.773	9.614	3.156
OV90	ovary	0.632	21.138	15.284	39.037
EFO27	ovary	0.644	0.143	1.167	2.878
PEO1	ovary	0.700	171.317	12.109	5.061
IOSE397	ovary	0.739	13.475	15.361	31.027
UWB1289	ovary	0.759	112.312	6.157	15.633
OVCAR8	ovary	0.912	49.346	11.920	40.389
IOSE523	ovary	0.917	39.456	18.871	115.658
OV56	ovary	0.931	16.693	125.551	149.546
SW626	ovary	1.101	458.240	42.551	15.602
JHOS3	ovary	1.175	158.283	6.629	10.660
RMGI	ovary	1.181		1.148	7.213
KURAMOCHI	ovary	1.266	146.168	7.452	13.813

ES2	ovary	2.117	17.064	32.640	190.432
CAOV4	ovary	2.447	2522.859	9.581	70.036
OV17R	ovary	3.258	264.728	10.071	116.040
OAW28	ovary	3.310	319.672	7.302	25.801
EFO21	ovary	4.860	230.625	5.904	86.422
FUOV1	ovary	8.594	97.019	18.710	13.710
OVISE	ovary	12.683	1.937	10.274	6.007
OVKATE	ovary	15.181	884.431	77.475	33.846
DANG	pancreas	0.010	64.669	11.368	6.222
PATU8902	pancreas	0.152	27.229	20.605	5.439
PANC0403	pancreas	0.262	962.413	12.519	37.257
HPAFII	pancreas	0.266	17.500	5.242	8.527
HUPT4	pancreas	0.280	1013.266	3.862	3.354
PATU8988T	pancreas	0.315	10.945	3.835	21.494
PL18	pancreas	0.357	22.185	5.523	182.578
KP1N	pancreas	0.377	148.669	26.152	11.641
MZ1PC	pancreas	0.529	88.110	4.722	7.128
KP4	pancreas	0.541	19.973	11.190	114.534
PANC0813	pancreas	0.704	230.069	11.302	12.617
PL4	pancreas	0.710	1697.115	40.212	19.526
MIAPACA2	pancreas	0.791	28.403	37.333	10.061
PANC0327	pancreas	1.006	367.561	2.237	44.039
CFPAC1	pancreas	1.039	1172.646	7.431	11.042
BXPC3	pancreas	1.074	43.212	11.419	37.624
SUIT2	pancreas	1.134	124.814	68.946	33.405
SU8686	pancreas	1.385	250.832	56.222	28.911
HS766T	pancreas	1.461	51.291	7.558	31.134
SW1990	pancreas	1.465	826.712	19.723	147.787
KP3	pancreas	1.925	68.062	47.284	25.682
PANC0203	pancreas	1.955	251.001	13.962	5.031
PANC1005	pancreas	2.240	89.986	7.403	24.290
HUPT3	pancreas	2.552	89.201	36.787	14.929
KP2	pancreas	3.782	110.477	14.845	43.642
HPAC	pancreas	4.461	136.253	9.548	25.648
PSN1	pancreas	5.657	194.259	140.792	581.005
QGP1	pancreas	6.622	3079.126	153.848	85.934
YAPC	pancreas	8.175	4768.423	37.662	24.014
CAPAN1	pancreas	17.872	273.699	37.798	85.208
LNCAPCLONEFGC	prostate	0.147	0.181	0.140	14.662
DU145	prostate	0.373	18.364	0.166	14.380
BPH1	prostate	0.506	3.456	1.843	3.542
22RV1	prostate	0.583	50.516	13.911	16.203
PC3	prostate	0.719	83.363	8.630	28.440
VCAP	prostate	4.699	37.402	24.426	13.680
PWR1E	prostate	7.812	21.497	19.945	24.784
MZ2MEL	skin	0.040	720.101	8.900	5.502
IGR37	skin	0.086	5.048	0.331	2.933

WM1158	skin	0.134	5.525	0.522	7.082
IPC298	skin	0.252	26.237	39.492	24.660
COLO800	skin	0.280	75.956	15.560	9.807
SKMEL5	skin	0.291	29.750	16.871	27.230
A375	skin	0.300	13.741	10.076	9.214
LOXIMVI	skin	0.304	1298.818	4.131	6.952
RPMI7951	skin	0.308	4.670	4.453	13.447
GAK	skin	0.317	322.245	19.521	36.043
CHL1	skin	0.380	64.497	1.635	11.825
MELHO	skin	0.403	6.109	0.237	58.395
ISTMEL1	skin	0.404	11.807	19.877	26.292
CP66MEL	skin	0.411	358.521	28.044	12.567
G361	skin	0.418	24.735	14.473	70.138
SH4	skin	0.477	52.148	1.859	131.478
WM793B	skin	0.481	14.397	19.745	872.790
MEWO	skin	0.499	34.236	14.302	61.589
GMEL	skin	0.525		11.374	23.796
A431	skin	0.548	72.780	26.110	32.175
MMACSF	skin	0.581		7.916	11.227
WM35	skin	0.586	32.400	26.015	14.892
MZ7MEL	skin	0.599	13.307	38.816	302.712
MELJUSO	skin	0.613	24.208	4.486	19.327
A388	skin	0.633	50.230	4.576	2.375
UACC62	skin	0.703	12.578	1.661	14.987
A101D	skin	0.760	23.475	12.408	149.749
HT144	skin	0.781	7.264	11.763	22.780
RVH421	skin	0.810	3.526	11.671	23.720
CP50MELB	skin	0.816	2.878	10.920	6.502
451LU	skin	0.829	15.389	246.178	396.711
SKMEL28	skin	0.897	11.279	10.890	11.470
IGR1	skin	0.973	7.299	127.562	367.335
K2	skin	0.975	44.002	21.058	15.457
HS939T	skin	1.004	17.245	5.077	15.284
MCC13	skin	1.038	2457.314	52.062	132.219
SKMEL2	skin	1.123	25.202	16.469	14.001
WM1552C	skin	1.215	45.480	46.331	48.594
SKMEL30	skin	1.334	91.684	134.448	27.060
DJM1	skin	1.342	840.840	128.530	54.566
SKMEL24	skin	1.418	18.285	8.566	217.463
WM278	skin	1.495	22.501	10.369	14.342
COLO792	skin	1.559	23.104	15.395	21.843
COLO679	skin	1.571	18.347	12.259	37.886
HS940T	skin	1.704	27.894	29.921	51.750
HMVII	skin	1.948	60.435	23.541	20.416
M14	skin	1.976	27.101	11.349	48.646
WM115	skin	2.174	6.256	8.840	27.125
MCC26	skin	2.664	71.870	85.420	31.584

C32	skin	2.746	14.327	18.228	71.122
UACC257	skin	3.592	32.108	21.218	72.822
COLO829	skin	3.694	219.655	42.510	59.667
SKMEL1	skin	4.419	528.869	274.229	767.592
COLO783	skin	4.617		20.420	28.183
SKMEL31	skin	14.195	26.886	29.638	362.200
LB2518MEL	skin	16.587	212.466	25.626	163.766
LB373MELD	skin	18.816	728.739	26.357	157.355
VMRCMELG	skin	29.055	18.527	14.560	14.682
RH1	soft tissue	0.271	16.678	40.308	3.761
MFHINO	soft tissue	0.743	11.834	14.737	59.278
VAESBJ	soft tissue	1.436	11.545	167.665	383.899
HEMCSS	soft tissue	1.468	4.376	17.376	1.649
SW872	soft tissue (fat)	0.430	8.035	10.831	5.682
HS633T	soft tissue (fibrous)	0.190	29.149	6.741	10.718
HT1080	soft tissue (fibrous)	0.581	8.704	104.351	28.848
GCT	soft tissue (fibrous)	0.614	3.105	16.674	249.955
SW684	soft tissue (fibrous)	10.565	10.073	7.111	43.497
SW982	soft tissue (synovial)	0.744	3.979	7.575	14.125
SNU5	stomach	0.138	20.353	3.130	21.879
FU97	stomach	0.180	3.138	0.740	11.645
TMK1	stomach	0.213	0.981	2.375	9.744
HSC39	stomach	0.230	1.434	11.054	1.590
NUGC3	stomach	0.290	122.060	12.357	60.337
2313287	stomach	0.293	1.945	1.367	7.041
ECC10	stomach	0.293	7.888	10.476	39.573
GT3TKB	stomach	0.440	6.304	4.203	69.441
N87	stomach	0.469	17.578	1.761	5.946
SKGT2	stomach	0.529	27.052	23.434	90.158
SCH	stomach	0.747	41.672	6.937	41.047
MKN1	stomach	0.781	56.205	7.559	5.342
HS746T	stomach	0.870	223.140	9.372	69.943
AGS	stomach	0.880	10.894	85.626	1.958
MKN45	stomach	1.243	178.076	85.192	45.731
KATOIII	stomach	1.313	3.007	276.018	21.747
SNU16	stomach	1.440	41.353	15.808	35.807
MKN28	stomach	2.074	1392.734	59.782	31.255
NUGC4	stomach	2.234	4807.719	10.107	29.854
MKN7	stomach	2.413	101.675	16.953	33.544
RERFGC1B	stomach	2.530	96.260	34.514	51.844
OCUM1	stomach	6.441	27.735	96.451	156.799
IM95	stomach	6.497	1.865	15.489	5.612
TGBC11TKB	stomach	7.350	462.319	145.216	24.384
SNU1	stomach	8.023	1235.652	261.327	814.703
RF48	stomach	20.407	182.653	37.915	101.897
K5	thyroid	0.174	0.531	3.026	1.244
BHT101	thyroid	0.180	11.871	1.849	8.171

CAL62	thyroid	0.184	84.066	46.376	17.649
HTCC3	thyroid	0.233	22.448	3.827	17.359
BCPAP	thyroid	0.289	22.884	9.001	141.268
IHH4	thyroid	0.330	46.909	8.836	67.886
8305C	thyroid	0.406	52.796	5.353	10.075
S117	thyroid	0.642	49.235	6.567	156.081
RO82W1	thyroid	0.711	18.229	22.079	21.183
ASH3	thyroid	1.833	61.863	17.443	17.301
8505C	thyroid	2.672	52.210	95.468	45.510
FTC133	thyroid	3.378	6.658	24.517	670.328
TT2609C02	thyroid	21.691	50.018	109.627	945.991
NTERASCLD1	urogenital	0.101	17.951	1.276	7.078
CAL39	urogenital	0.155	4.475	11.845	5.026
SW962	urogenital	0.170	17.296	0.612	9.224
SW954	urogenital	0.517	1.440	8.769	2.641
NEC8	urogenital	0.951	112.584	16.510	31.544
JEG3	urogenital	1.706	109.409	9.393	184.257
JAR	urogenital	9.274	9.406	2.029	231.502
MFE296	uterus	0.110	0.009	5.737	14.135
MFE280	uterus	0.122	83.062	4.484	3.283
AN3CA	uterus	0.181	0.868	3.826	5.084
SKUT1	uterus	0.243	83.812	10.065	30.093
MESSA	uterus	0.360	14.220	13.072	4.612
SKN	uterus	0.554	2.129	0.620	29.560
639V	uterus	0.692	825.328	5.471	7.012
MFE319	uterus	0.821	0.236	13.934	63.340
COLO684	uterus	0.824	0.278	5.172	18.429
HEC1	uterus	0.871	32.577	205.411	27.054
SNGM	uterus	0.993	11.404	5.462	2.022
ISHIKAWAHERAKLIO02ER	uterus	1.054	2.977	5.675	7.335
KLE	uterus	1.762	44.797	4.243	100.305
RL952	uterus	2.002		34.574	637.420
ESS1	uterus	2.750	82.869	36.667	7.093
EN	uterus	9.377	10.564	113.612	166.854

*NB- There are 43 cell lines where an IC_{50} value was not generated for AZD5363, this is due to a technical error from the screen.

Supplementary Table 2: Sanger cancer cell line pharmacology results and subtype classification for hematological malignancies

Cell Line	Tissue	AZD2014 (IC_{50} uM)	AZD5363 (IC_{50} uM)	AZD8186 (IC_{50} uM)	AZD8835 (IC_{50} uM)	Subtype	Further Classification
RCHACV	blood/lymph	0.255	0.910	0.655	0.715	acute lymphoblastic leukemia (ALL) (B-cell)	acute lymphoblastic leukemia (ALL) (B-cell)
GRST	blood/lymph	0.052	1.208	3.481	1.874	acute lymphoblastic leukemia (ALL) (B-cell)	acute lymphoblastic leukemia (ALL) (B-cell)
ALLPO	blood/lymph	9.273	1.611	50.405	147.828	acute lymphoblastic leukemia (ALL) (B-cell)	acute lymphoblastic leukemia (ALL) (B-cell)
KARPAS231	blood/lymph	0.763	1.616	3.168	11.618	acute lymphoblastic leukemia (ALL) (B-cell)	acute lymphoblastic leukemia (ALL) (B-cell)
REH	blood/lymph	0.149	1.836	0.850	0.666	acute lymphoblastic leukemia (ALL) (B-cell)	acute lymphoblastic leukemia (ALL) (B-cell)
NALM6	blood/lymph	0.691	2.052	3.270	3.536	acute lymphoblastic leukemia (ALL) (B-cell)	acute lymphoblastic leukemia (ALL) (B-cell)
RS411	blood/lymph	1.173	2.501	34.067	98.665	acute lymphoblastic leukemia (ALL) (B-cell)	acute lymphoblastic leukemia (ALL) (B-cell)
HAL01	blood/lymph	0.222	5.195	0.660	0.250	acute lymphoblastic leukemia (ALL) (B-cell)	acute lymphoblastic leukemia (ALL) (B-cell)
697	blood/lymph	0.069	5.329	1.991	1.396	acute lymphoblastic leukemia (ALL) (B-cell)	acute lymphoblastic leukemia (ALL) (B-cell)
WIL2NS	blood/lymph	0.461	36.669	31.636	23.199	acute lymphoblastic leukemia (ALL) (B-cell)	acute lymphoblastic leukemia (ALL) (B-cell)
BALL1	blood/lymph	5.440	74.328	51.484	634.828	acute lymphoblastic leukemia (ALL) (B-cell)	acute lymphoblastic leukemia (ALL) (B-cell)
MHHCALL2	blood/lymph	27.819	169.518	133.014	739.901	acute lymphoblastic leukemia (ALL) (B-cell)	acute lymphoblastic leukemia (ALL) (B-cell)
VAL	blood/lymph	0.448	241.688	14.824	33.459	acute lymphoblastic leukemia (ALL) (B-cell)	acute lymphoblastic leukemia (ALL) (B-cell)
MN60	blood/lymph	0.379	242.568	37.411	28.227	acute lymphoblastic leukemia (ALL) (B-cell)	acute lymphoblastic leukemia (ALL) (B-cell)
ROS50	blood/lymph	43.499	590.362	118.654	443.063	acute lymphoblastic leukemia (ALL) (B-cell)	acute lymphoblastic leukemia (ALL) (B-cell)
KOPN8	blood/lymph	0.473		41.143	266.524	acute lymphoblastic leukemia (ALL) (B-cell)	acute lymphoblastic leukemia (ALL) (B-cell)
MOLT16	blood/lymph	0.186	0.025	43.151	8.730	acute lymphoblastic leukemia (ALL) (T-cell)	acute lymphoblastic leukemia (ALL) (T-cell)
JRT3T35	blood/lymph	0.079	0.124	7.610	96.813	acute lymphoblastic leukemia (ALL) (T-cell)	acute lymphoblastic leukemia (ALL) (T-cell)
LOUCY	blood/lymph	0.197	0.147	139.459	3.362	acute lymphoblastic leukemia (ALL) (T-cell)	acute lymphoblastic leukemia (ALL) (T-cell)
KE37	blood/lymph	0.424	0.173	164.312	337.216	acute lymphoblastic leukemia (ALL) (T-cell)	acute lymphoblastic leukemia (ALL) (T-cell)
CCRFCEM	blood/lymph	0.038	0.231	13.779	6.943	acute lymphoblastic leukemia (ALL) (T-cell)	acute lymphoblastic leukemia (ALL) (T-cell)
MOLT4	blood/lymph	0.046	0.277	0.530	0.811	acute lymphoblastic leukemia (ALL) (T-cell)	acute lymphoblastic leukemia (ALL) (T-cell)
KARPAS45	blood/lymph	0.685	2.175	1.906	23.922	acute lymphoblastic leukemia (ALL) (T-cell)	acute lymphoblastic leukemia (ALL) (T-cell)
ATN1	blood/lymph	0.342	2.783	6.219	2.427	acute lymphoblastic leukemia (ALL) (T-cell)	adult T-cell leukaemia
RPMI8402	blood/lymph	0.075	3.659	0.218	1.100	acute lymphoblastic leukemia (ALL) (T-cell)	acute lymphoblastic leukemia (ALL) (T-cell)
PF382	blood/lymph	4.301	4.856	170.133	111.204	acute lymphoblastic leukemia (ALL) (T-cell)	acute lymphoblastic leukemia (ALL) (T-cell)
SUPT1	blood/lymph	8.984	5.558	364.567	294.388	acute lymphoblastic leukemia (ALL) (T-cell)	acute lymphoblastic leukemia (ALL) (T-cell)
JURKAT	blood/lymph	0.271	7.445	26.197	11.429	acute lymphoblastic leukemia (ALL) (T-cell)	acute lymphoblastic leukemia (ALL) (T-cell)
BE13	blood/lymph	0.198	11.073	305.799	141.762	acute lymphoblastic leukemia (ALL) (T-cell)	acute lymphoblastic leukemia (ALL) (T-cell)
P12ICHIKAWA	blood/lymph	3.347	18.252	149.770	83.434	acute lymphoblastic leukemia (ALL) (T-cell)	acute lymphoblastic leukemia (ALL) (T-cell)

MOLT13	blood/lymph	0.228	34.297	58.799	276.863	acute lymphoblastic leukemia (ALL) (T-cell)	acute lymphoblastic leukemia (ALL) (T-cell)
DND41	blood/lymph	0.912	40.063	24.543	87.633	acute lymphoblastic leukemia (ALL) (T-cell)	acute lymphoblastic leukemia (ALL) (T-cell)
ALLSIL	blood/lymph	0.484		6.723	4.755	acute lymphoblastic leukemia (ALL) (T-cell)	acute lymphoblastic leukemia (ALL) (T-cell)
P30OHK	blood/lymph	0.049	8.710	0.287	0.222	acute lymphoblastic leukemia (ALL)_other	acute lymphoblastic leukemia (ALL) (not otherwise specified)
YT	blood/lymph	4.548	53.347	136.455	709.283	acute lymphoblastic leukemia (ALL)_other	acute lymphoblastic leukemia (ALL) (not otherwise specified)
LC41	blood/lymph	0.835	191.636	52.465	171.039	acute lymphoblastic leukemia (ALL)_other	acute lymphoblastic leukemia (ALL) (not otherwise specified)
KASUMI1	blood/lymph	0.153	0.499	0.911	0.437	acute myelogenous leukemia (AML)	acute myelogenous leukemia (AML)
MOLM13	blood/lymph	0.089	0.659	5.041	1.727	acute myelogenous leukemia (AML)	acute myelogenous leukemia (AML)
MC1010	blood/lymph	0.270	0.686	0.362	0.629	acute myelogenous leukemia (AML)	acute myelogenous leukemia (AML)
EOL1	blood/lymph	0.168	0.905	24.014	5.560	acute myelogenous leukemia (AML)	acute myelogenous leukemia (AML)
PL21	blood/lymph	0.653	1.083	7.241	2.389	acute myelogenous leukemia (AML)	acute myelogenous leukemia (AML)
MOLM16	blood/lymph	0.050	1.094	0.341	0.989	acute myelogenous leukemia (AML)	acute myelogenous leukemia (AML)
SKM1	blood/lymph	0.722	1.453	1.928	1.776	acute myelogenous leukemia (AML)	acute myelogenous leukemia (AML)
MONOMAC6	blood/lymph	0.166	1.455	4.420	2.518	acute myelogenous leukemia (AML)	acute myelogenous leukemia (AML)
MV411	blood/lymph	0.121	1.908	13.662	4.072	acute myelogenous leukemia (AML)	acute myelogenous leukemia (AML)
CTV1	blood/lymph	0.771	2.899	18.493	22.438	acute myelogenous leukemia (AML)	acute myelogenous leukemia (AML)
P31FUJ	blood/lymph	1.100	3.207	184.763	16.751	acute myelogenous leukemia (AML)	acute myelogenous leukemia (AML)
NOMO1	blood/lymph	0.367	4.286	13.741	4.725	acute myelogenous leukemia (AML)	acute myelogenous leukemia (AML)
KMOE2	blood/lymph	0.258	6.687	25.880	11.267	acute myelogenous leukemia (AML)	acute myelogenous leukemia (AML)
OCIAML3	blood/lymph	0.394	7.274	8.069	2.138	acute myelogenous leukemia (AML)	acute myelogenous leukemia (AML)
HL60	blood/lymph	23.531	8.015	31.594	47.828	acute myelogenous leukemia (AML)	acute myelogenous leukemia (AML)
NKM1	blood/lymph	0.135	11.410	3.911	2.997	acute myelogenous leukemia (AML)	acute myelogenous leukemia (AML)
ML2	blood/lymph	0.386	12.464	1.999	5.795	acute myelogenous leukemia (AML)	acute myelogenous leukemia (AML)
KY821	blood/lymph	43.582	13.022	12.869	18.201	acute myelogenous leukemia (AML)	acute myelogenous leukemia (AML)
NB4	blood/lymph	0.510	18.251	4.907	2.490	acute myelogenous leukemia (AML)	acute myelogenous leukemia (AML)
THP1	blood/lymph	10.825	22.681	16.738	25.681	acute myelogenous leukemia (AML)	acute myelogenous leukemia (AML)
QIMRWIL	blood/lymph	0.491	31.746	5.716	25.875	acute myelogenous leukemia (AML)	acute myelogenous leukemia (AML)
SET2	blood/lymph	1.359	36.027	158.881	35.886	acute myelogenous leukemia (AML)	acute myelogenous leukemia (AML)
OCIAML2	blood/lymph	0.264	42.213	31.289	27.789	acute myelogenous leukemia (AML)	acute myelogenous leukemia (AML)
CESS	blood/lymph	0.525	59.284	3.623	8.225	acute myelogenous leukemia (AML)	acute myelogenous leukemia (AML)
CMK	blood/lymph	0.768	105.408	68.138	42.115	acute myelogenous leukemia (AML)	acute myelogenous leukemia (AML)

KG1	blood/lymph	0.151	113.113	32.798	9.277	acute myelogenous leukemia (AML)	acute myelogenous leukemia (AML)
OCIAML5	blood/lymph	1.705	386.588	5.672	2.437	acute myelogenous leukemia (AML)	acute myelogenous leukemia (AML)
OCIM1	blood/lymph	1.259	450.151	222.589	847.650	acute myelogenous leukemia (AML)	acute myelogenous leukemia (AML)
GDM1	blood/lymph	0.218	1027.702	2.986	1.282	acute myelogenous leukemia (AML)	acute myelogenous leukemia (AML)
HEL	blood/lymph	1.023	1370.458	306.918	600.188	acute myelogenous leukemia (AML)	acute myelogenous leukemia (AML)
ME1	blood/lymph	7.599	2814.551	191.303	1974.787	acute myelogenous leukemia (AML)	acute myelogenous leukemia (AML)
RAMOS2G64C10	blood/lymph	0.044	1.517	14.669	287.287	Burkitt lymphoma (B-cell)	Burkitt lymphoma (B-cell)
BL41	blood/lymph	0.544	3.454	22.007	70.892	Burkitt lymphoma (B-cell)	Burkitt lymphoma (B-cell)
DG75	blood/lymph	0.172	4.847	54.735	155.821	Burkitt lymphoma (B-cell)	Burkitt lymphoma (B-cell)
GA10	blood/lymph	0.046	5.785	3.171	13.316	Burkitt lymphoma (B-cell)	Burkitt lymphoma (B-cell)
P32ISH	blood/lymph	0.544	8.026	6.395	9.086	Burkitt lymphoma (B-cell)	Burkitt lymphoma (B-cell)
RL	blood/lymph	2.182	12.663	4.445	37.120	Burkitt lymphoma (B-cell)	Burkitt lymphoma (B-cell)
SUPB8	blood/lymph	2.596	15.504	91.747	960.551	Burkitt lymphoma (B-cell)	Burkitt lymphoma (B-cell)
DAUDI	blood/lymph	0.179	39.470	5.957	10.877	Burkitt lymphoma (B-cell)	Burkitt lymphoma (B-cell)
EB2	blood/lymph	3.885	53.938	31.059	70.204	Burkitt lymphoma (B-cell)	Burkitt lymphoma (B-cell)
NAMALWA	blood/lymph	0.757	63.992	15.219	7.873	Burkitt lymphoma (B-cell)	Burkitt lymphoma (B-cell)
ST486	blood/lymph	1.365	112.482	225.841	1748.013	Burkitt lymphoma (B-cell)	Burkitt lymphoma (B-cell)
CA46	blood/lymph	0.115	154.631	8.848	12.762	Burkitt lymphoma (B-cell)	Burkitt lymphoma (B-cell)
JIYOYEP2003	blood/lymph	0.126	214.340	17.036	24.798	Burkitt lymphoma (B-cell)	Burkitt lymphoma (B-cell)
RAJI	blood/lymph	0.181	716.889	58.906	157.034	Burkitt lymphoma (B-cell)	Burkitt lymphoma (B-cell)
EB3	blood/lymph	0.083	891.047	25.739	104.271	Burkitt lymphoma (B-cell)	Burkitt lymphoma (B-cell)
BL70	blood/lymph	0.121		3.630	7.916	Burkitt lymphoma (B-cell)	Burkitt lymphoma (B-cell)
EHEB	blood/lymph	7.494	2.206	0.350	27.656	chronic lymphocytic leukemia (CLL) (B-cell)	chronic lymphocytic leukemia (CLL) (B-cell)
MEC1	blood/lymph	0.062	5.479	0.436	0.320	chronic lymphocytic leukemia (CLL) (B-cell)	chronic lymphocytic leukemia (CLL) (B-cell)
JVM3	blood/lymph	0.405	6.837	22.366	19.983	chronic lymphocytic leukemia (CLL) (B-cell)	chronic lymphocytic leukemia (CLL) (B-cell), prolymphocytic leukemia (PLL) (B-cell)
EM2	blood/lymph	0.055	2.082	8.070	8.349	chronic myelogenous leukemia (CML)	chronic myelogenous leukemia (CML)
BV173	blood/lymph	0.385	2.686	171.246	16.904	chronic myelogenous leukemia (CML)	chronic myelogenous leukemia (CML)
RPMI8866	blood/lymph	0.100	3.015	0.266	0.635	chronic myelogenous leukemia (CML)	chronic myelogenous leukemia (CML)
JURLMK1	blood/lymph	0.827	6.538	90.881	202.677	chronic myelogenous leukemia (CML)	chronic myelogenous leukemia (CML)
KU812	blood/lymph	0.178	7.788	48.495	1086.897	chronic myelogenous leukemia (CML)	chronic myelogenous leukemia (CML)
K562	blood/lymph	0.499	53.881	135.178	773.969	chronic myelogenous leukemia (CML)	chronic myelogenous leukemia (CML)

CMLT1	blood/lymph	0.584	130.211	152.273	942.283	chronic myelogenous leukemia (CML)	chronic myelogenous leukemia (CML)
MEG01	blood/lymph	0.267	141.030	123.585	119.371	chronic myelogenous leukemia (CML)	chronic myelogenous leukemia (CML)
LAMA84	blood/lymph	4.968	190.251	60.663	580.681	chronic myelogenous leukemia (CML)	chronic myelogenous leukemia (CML)
KCL22	blood/lymph	0.076		28.319	36.555	chronic myelogenous leukemia (CML)	chronic myelogenous leukemia (CML)
WSUDLCL2	blood/lymph	536.807	0.003	37.875	781.327	diffuse large B-cell lymphoma (DLBCL)	diffuse large B-cell lymphoma (DLBCL)
OCILY7	blood/lymph	0.249	0.076	2.015	7.747	diffuse large B-cell lymphoma (DLBCL)	diffuse large B-cell lymphoma (DLBCL)
SUDHL4	blood/lymph	0.893	0.238	1.012	458.613	diffuse large B-cell lymphoma (DLBCL)	diffuse large B-cell lymphoma (DLBCL)
SUDHL5	blood/lymph	0.038	0.300	0.036	0.187	diffuse large B-cell lymphoma (DLBCL)	diffuse large B-cell lymphoma (DLBCL)
WSUNHL	blood/lymph	0.185	0.344	0.149	0.247	diffuse large B-cell lymphoma (DLBCL)	diffuse large B-cell lymphoma (DLBCL)
SUDHL10	blood/lymph	2.805	0.395	12.216	23.354	diffuse large B-cell lymphoma (DLBCL)	diffuse large B-cell lymphoma (DLBCL)
SUDHL16	blood/lymph	0.178	0.505	3.235	1.285	diffuse large B-cell lymphoma (DLBCL)	diffuse large B-cell lymphoma (DLBCL)
FARAGE	blood/lymph	1044.822	0.839	286.282	1128.638	diffuse large B-cell lymphoma (DLBCL)	diffuse large B-cell lymphoma (DLBCL)
A4FUK	blood/lymph	0.128	0.855	10.424	14.779	diffuse large B-cell lymphoma (DLBCL)	diffuse large B-cell lymphoma (DLBCL)
HT	blood/lymph	0.852	1.174	6.688	23.004	diffuse large B-cell lymphoma (DLBCL)	diffuse large B-cell lymphoma (DLBCL)
KARPAS422	blood/lymph	5.090	2.809	1.839	25.690	diffuse large B-cell lymphoma (DLBCL)	diffuse large B-cell lymphoma (DLBCL)
CTB1	blood/lymph	1.134	11.693	16.675	307.773	diffuse large B-cell lymphoma (DLBCL)	diffuse large B-cell lymphoma (DLBCL)
OCILY19	blood/lymph	0.962	18.448	13.133	37.825	diffuse large B-cell lymphoma (DLBCL)	diffuse large B-cell lymphoma (DLBCL)
U698M	blood/lymph	0.138	29.797	21.165	88.321	diffuse large B-cell lymphoma (DLBCL)	diffuse large B-cell lymphoma (DLBCL)
TK	blood/lymph	0.757	29.973	20.978	63.742	diffuse large B-cell lymphoma (DLBCL)	diffuse large B-cell lymphoma (DLBCL)
SUDHL8	blood/lymph	0.918	70.804	64.530	270.072	diffuse large B-cell lymphoma (DLBCL)	diffuse large B-cell lymphoma (DLBCL)
A3KAW	blood/lymph	0.102	188.901	16.825	240.363	diffuse large B-cell lymphoma (DLBCL)	diffuse large B-cell lymphoma (DLBCL)
RCK8	blood/lymph	261.043	1901.977	139.477	474.596	diffuse large B-cell lymphoma (DLBCL)	diffuse large B-cell lymphoma (DLBCL)
SCI1	blood/lymph	0.323	3559.114	23.254	129.733	diffuse large B-cell lymphoma (DLBCL)	diffuse large B-cell lymphoma (DLBCL)
DB	blood/lymph	0.124		14.954	175.601	diffuse large B-cell lymphoma (DLBCL)	diffuse large B-cell lymphoma (DLBCL)
L1236	blood/lymph	0.395	3.133	16.137	17.078	Hodgkin lymphoma	Hodgkin lymphoma
RPMI6666	blood/lymph	2.216	4.096	10.551	14.758	Hodgkin lymphoma	Hodgkin lymphoma
SUPHD1	blood/lymph	0.885	5.543	31.992	73.944	Hodgkin lymphoma	Hodgkin lymphoma
KMH2	blood/lymph	0.802	23.928	41.132	64.305	Hodgkin lymphoma	Hodgkin lymphoma
HDLM2	blood/lymph	7.185	34.375	73.058	165.795	Hodgkin lymphoma	Hodgkin lymphoma
L540	blood/lymph	0.188	61.509	43.411	10.889	Hodgkin lymphoma	Hodgkin lymphoma
HDMYZ	blood/lymph	0.298	118.538	17.411	35.880	Hodgkin lymphoma	Hodgkin lymphoma
L428	blood/lymph	2.298	1592.791	133.557	408.307	Hodgkin lymphoma	Hodgkin lymphoma
HS445	blood/lymph	6.722		5.603	1.818	Hodgkin lymphoma	Hodgkin lymphoma

L363	blood/lymph	0.051	0.273	1.993	0.525	leukemia_other	plasma cell leukemia (PCL)
KARPAS620	blood/lymph	0.274	0.473	10.986	6.115	leukemia_other	plasma cell leukemia (PCL)
MOT	blood/lymph	7.226	7.381	32.218	47.028	leukemia_other	hairy cell leukemia (HCL) (T-cell)
JJN3	blood/lymph	0.358	9.531	189.519	78.551	leukemia_other	plasma cell leukemia (PCL)
SKMM2	blood/lymph	0.260	23.948	23.980	5.197	leukemia_other	plasma cell leukemia (PCL)
MLMA	blood/lymph	3.730	58.228	7.291	3.691	leukemia_other	hairy cell leukemia (HCL) (B-cell)
HC1	blood/lymph	0.146	92.931	3.848	3.709	leukemia_other	hairy cell leukemia (HCL)
TUR	blood/lymph	0.076	0.332	4.761	4.194	lymphoma_other	histiocytic lymphoma
SR	blood/lymph	0.304	2.009	20.767	9.299	lymphoma_other	lymphoma (not otherwise specified)
JVM2	blood/lymph	0.292	5.080	0.834	2.045	lymphoma_other	mantle cell lymphoma (MCL)
HH	blood/lymph	0.015	15.159	0.075	0.243	lymphoma_other	cutaneous T-cell lymphoma (CTCL)
H9	blood/lymph	0.285	42.140	74.904	36.009	lymphoma_other	cutaneous T-cell lymphoma (CTCL)
KARPAS299	blood/lymph	0.820	57.146	83.847	165.436	lymphoma_other	anaplastic large cell lymphoma (ALCL) (T-cell)
JM1	blood/lymph	2.418	79.891	35.655	46.144	lymphoma_other	lymphoma (B-cell) (not otherwise specified)
SUPM2	blood/lymph	0.047	110.527	2.201	3.174	lymphoma_other	anaplastic large cell lymphoma (ALCL) (T-cell)
MC116	blood/lymph	0.851	892.108	20.367	86.809	lymphoma_other	lymphoma (B-cell) (not otherwise specified)
DEL	blood/lymph	0.089	2024.822	8.091	148.056	lymphoma_other	anaplastic large cell lymphoma (ALCL) (T-cell)
H929	blood/lymph	0.043	0.003	0.729	0.285	multiple myeloma (MM)	multiple myeloma (MM)
MCCAR	blood/lymph	0.135	0.146	179.217	13.090	multiple myeloma (MM)	multiple myeloma (MM)
OPM2	blood/lymph	1.621	2.499	139.191	387.894	multiple myeloma (MM)	multiple myeloma (MM)
MOLP8	blood/lymph	0.160	2.535	3.359	3.786	multiple myeloma (MM)	multiple myeloma (MM)
LP1	blood/lymph	1.432	16.174	114.760	289.315	multiple myeloma (MM)	multiple myeloma (MM)
RPMI8226	blood/lymph	0.121	20.862	9.733	120.000	multiple myeloma (MM)	multiple myeloma (MM)
EJM	blood/lymph	0.753	33.121	23.746	111.305	multiple myeloma (MM)	multiple myeloma (MM)
KMS11	blood/lymph	0.267	295.708	7.989	4.970	multiple myeloma (MM)	multiple myeloma (MM)
IM9	blood/lymph	1.184	1064.479	348.479	159.194	multiple myeloma (MM)	multiple myeloma (MM)
ARH77	blood/lymph	0.258	1821.187	7.327	10.417	multiple myeloma (MM)	multiple myeloma (MM)
U266	blood/lymph	3.330		40.322	348.119	multiple myeloma (MM)	multiple myeloma (MM)
AMO1	blood/lymph	0.119	11.791	6.788	6.034	myeloma_other	plasmacytoma
NUDUL1	blood/lymph	7.095	0.355	17.071	37.439	non-Hodgkin lymphoma (NHL) (B-cell)	non-Hodgkin lymphoma (NHL) (B-cell)
CROAP2	blood/lymph	0.101	1.363	4.170	2.806	non-Hodgkin lymphoma (NHL) (B-cell)	non-Hodgkin lymphoma (NHL) (B-cell), primary effusion lymphoma (PEL)

JEKO1	blood/lymph	0.098	2.718	1.402	1.966	non-Hodgkin lymphoma (NHL) (B-cell)	non-Hodgkin lymphoma (NHL) (B-cell)
KARPAS1106P	blood/lymph	1.193	5.811	15.941	315.771	non-Hodgkin lymphoma (NHL) (B-cell)	non-Hodgkin lymphoma (NHL) (B-cell)
MHHPREB1	blood/lymph	0.049	6.218	1.450	6.082	non-Hodgkin lymphoma (NHL) (B-cell)	non-Hodgkin lymphoma (NHL) (B-cell)
CROAP3	blood/lymph	0.041	7.219	45.969	68.326	non-Hodgkin lymphoma (NHL) (B-cell)	non-Hodgkin lymphoma (NHL) (B-cell), primary effusion lymphoma (PEL)
JSC1	blood/lymph	0.695	13.649	246.620	189.798	non-Hodgkin lymphoma (NHL) (B-cell)	non-Hodgkin lymphoma (NHL) (B-cell), primary effusion lymphoma (PEL)
GRANTA519	blood/lymph	2.801	30.580	114.589	54.760	non-Hodgkin lymphoma (NHL) (B-cell)	non-Hodgkin lymphoma (NHL) (B-cell)
SLVL	blood/lymph	4.966	59.456	36.874	61.930	non-Hodgkin lymphoma (NHL) (B-cell)	non-Hodgkin lymphoma (NHL) (B-cell), splenic marginal zone lymphoma (SMZL)
BC1	blood/lymph	0.091	76.186	26.256	58.809	non-Hodgkin lymphoma (NHL) (B-cell)	non-Hodgkin lymphoma (NHL) (B-cell), primary effusion lymphoma (PEL)

Supplementary Table 3: Synergy scores from the T-ALL PI3K intra-pathway combination screen

	CCRFCEM	JRT3T35	JURKAT	MOLT4	PF382	RPMI8402	SUPT1
AZD2014_AZD5363	−2.3	1.46	1.89	−4.38	−0.55	−0.44	1.24
AZD8186_AZD2014	−0.93	0.13	2.1	−0.04	−0.16	0.84	−0.7
AZD8186_AZD5363	0.82	0.12	−0.26	−2.04	−0.29	−0.85	−3.17
AZD8186_AZD8835	0.09	0	0.54	1.19	0.3	−0.62	−3.61
AZD8835_AZD2014	1.69	−0.32	−0.89	−1.01	0.03	−0.42	−0.18
AZD8835_AZD5363	−0.64	−0.19	−1.28	−3.36	−0.11	−1.54	−3.32

A panel of T-ALL cell lines were treated with a dose response of different PI3K pathway inhibitors in a 8 × 8 dose response matrix. Live cell number was assessed after 5 days using a sytox green endpoint and synergy scores were generated using the Loewe model of additivity. Values represent the mean synergy score for each combination indicated (*n* = 2).

Supplementary Table 4: PI3K enzyme and cell IC_{50} (μM) data for PI3K compounds

	Enzyme Assay				Cell Assay			
	PI3Kα	PI3Kβ	PI3Kδ	PI3Kγ	PI3Kα-driven BT474	PI3Kβ-driven MDAMB468	PI3Kδ-driven JEKO	PI3Kγ-driven RAW264
AZD8186	0.045	0.001	0.006	0.223	0.725	0.003	0.017	> 1
AZD8835	0.006	0.431	0.006	0.090	0.090	3.555	0.049	0.532
BYL719	0.005	0.852	0.058	0.138	0.093	9.740	0.255	0.138
GDC0941	0.005	0.028	0.004	0.068	0.044	0.172	0.010	0.150
CAL101	0.715	0.260	0.004	0.072	12.130	0.638	0.004	2.333
IPI-145	0.182	0.021	< 0.001	0.006	0.775	0.098	< 0.001	0.103

Enzymes assayed by ADP Kinase Glo. Cell assays measured AKT phosphorylation. Data is geometric means of multiple IC_{50} determinations.
